# Probing the role of left inferior frontal gyrus in vocal motor control: Evidence from TMS and causal inference analysis

**DOI:** 10.1016/j.isci.2026.117790

**Published:** 2026-10-08

**Authors:** Yichen Chang, Qifan Guo, Shuzhi Zhao, Tingni Li, Xiuqin Wu, Dongxu Liu, Guangyan Dai, Xiaoxia Zhu, Xi Chen, Lan Wang, Peng Liu, Nan Yan, Hanjun Liu

**Affiliations:** 1 Department of Rehabilitation Medicine, The First Affiliated Hospital, Sun Yat-sen University, Guangzhou 510080, China; 2 CAS Key Laboratory of Human-Machine Intelligence-Synergy Systems, Shenzhen Institutes of Advanced Technology, Chinese Academy of Sciences, Shenzhen 518055, China; 3 Guangdong-Hong Kong-Macao Joint Laboratory of Human-Machine Intelligence-Synergy Systems, Shenzhen Institutes of Advanced Technology, Chinese Academy of Sciences, Shenzhen 518055, China; 4 Centre for Eye and Vision Research (CEVR), 17W Hong Kong Science Park, Hong Kong SAR 999077, China; 5 Guangdong Provincial Key Laboratory of Brain Function and Disease, Zhongshan School of Medicine, Sun Yat-sen University, Guangzhou 510080, China

**Keywords:** speech motor control, inferior frontal gyrus, auditory feedback, continuous theta burst stimulation, causal inference analysis

## Abstract

Beyond an established role in speech initiation and planning, the left inferior frontal gyrus (IFG) may contribute to sensorimotor control of speech production. We applied continuous theta burst stimulation (cTBS) over the anterior (aIFG) and posterior (pIFG) regions to investigate their roles in vocal motor control. A causal inference analysis (CIA) framework was developed to characterize source-space network organization. cTBS over both left aIFG and pIFG reduced vocal responses and enhanced P2 responses to pitch feedback perturbations, with no significant differences between two active stimulations. However, global dissimilarity analysis revealed greater P2-related topographic deviation from sham stimulation following aIFG stimulation than following pIFG stimulation. CIA further identified different source-space network patterns associated with aIFG and pIFG stimulation. These findings demonstrate the causal involvement of the left IFG in auditory-vocal integration and highlight the potential of CIA for characterizing distributed network organization underlying vocal motor control.

## Highlights

• cTBS over left aIFG and pIFG reduces vocal compensation for pitch perturbations
• cTBS over both IFG targets enhances P2 responses during vocal feedback control
• aIFG stimulation produces greater P2 topographic modulation relative to sham than pIFG stimulation
• Causal inference analysis identifies distinct network patterns for aIFG and pIFG stimulation

## Graphical abstract

**Figure:**
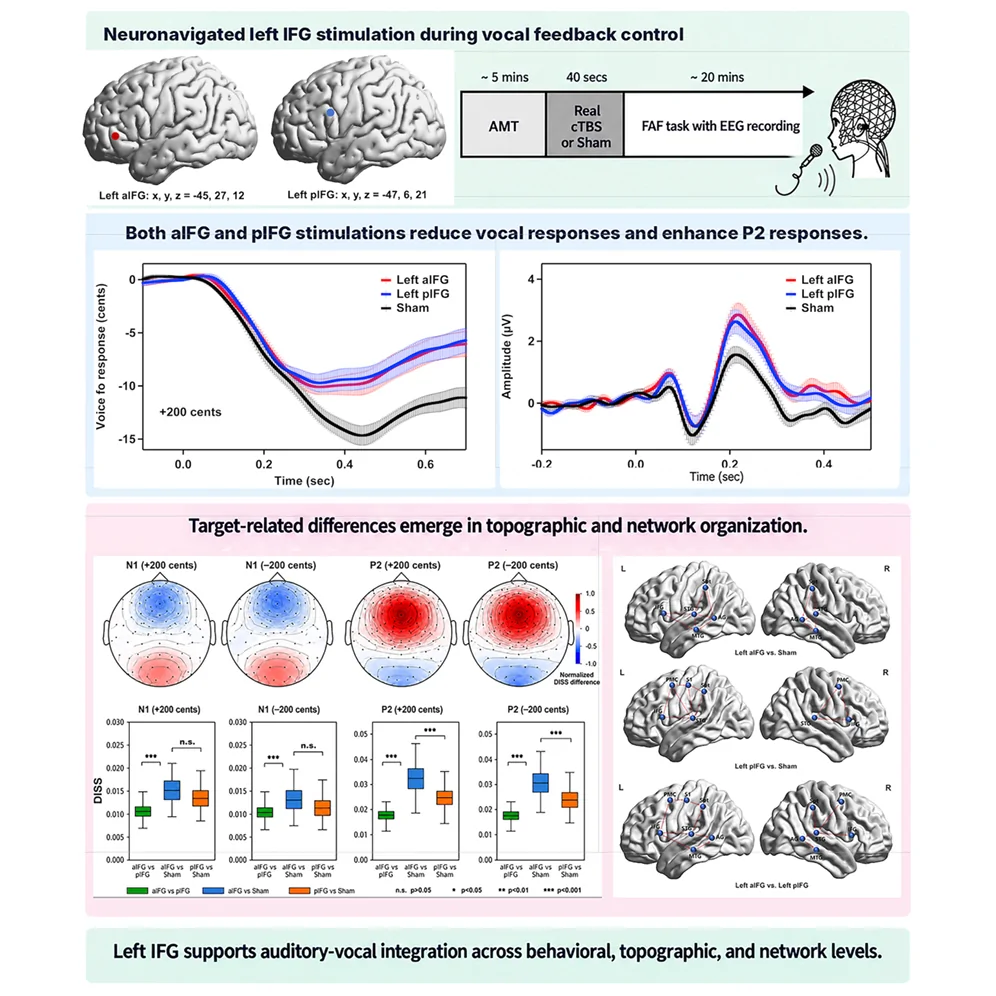

## Teaser

Neuroscience; Cognitive neuroscience

## Introduction

Speech production is a complex human behavior that relies heavily on precise integration between sensory feedback and motor commands to achieve accurate vocal output.^1^ Auditory feedback plays a central role in continuously monitoring vocal output errors^2^ and rapidly generating corrective vocal responses.^3^^,^^4^^,^^5^ While neuroimaging and electrophysiological studies have mapped a distributed cortico-subcortical network underlying auditory-motor integration,^6^^,^^7^^,^^8^^,^^9^^,^^10^^,^^11^^,^^12^ these findings are predominantly correlational, leaving open the causal contributions of individual brain regions within this network.

Central to the neural basis of speech production is the left inferior frontal gyrus (IFG), an important region that has been consistently implicated in speech processing.^13^ Computational models, such as the Directions Into Velocities of Articulators (DIVA) model^14^ and the dual stream model,^15^ propose that the left IFG is involved in translating auditory information into articulatory motor commands through feedforward and feedback mechanisms. Empirical studies further support these theoretical models by showing left IFG activation during speech planning, programming, and execution.^16^^,^^17^^,^^18^ However, the precise role of the left IFG in sensorimotor control of speech production, particularly via auditory feedback, remains insufficiently understood.

To date, there is mixed evidence regarding the involvement of the left IFG in auditory feedback control of vocal production. For example, structural equation modeling identified increased functional connectivity of the left IFG with the left superior temporal gyrus (STG) and left premotor cortex (PMC) in response to pitch perturbations in voice auditory feedback.^19^ More recently, applying high-definition transcranial alternating current stimulation (HD-tACS) over the left IFG at 6 or 70 Hz led to enhanced vocal and event-related potential (ERP) P2 responses to pitch perturbations.^20^ In contrast, several other studies reported absent left IFG activation when auditory feedback was unexpectedly perturbed.^7^^,^^10^^,^^11^ Such discrepancies may be related to the functional heterogeneity of the left IFG, which comprises anterior and posterior subregions with distinct structural and functional characteristics.^21^ Specifically, the left anterior IFG (aIFG) has been implicated in semantic processing and lexical retrieval,^22^^,^^23^ while the left posterior IFG (pIFG) is more closely linked to phonological processing and articulatory planning.^24^^,^^25^ Whether this functional heterogeneity extends to auditory-vocal integration, however, remains unclear. For example, damage to the left aIFG was significantly correlated with diminished vocal responses to pitch perturbations,^26^ whereas activation of the left pIFG has been identified in this feedback control process.^27^ These discrepancies suggest that the role of the left IFG in vocal feedback control may be shaped by its anatomical and functional heterogeneity, highlighting the need to examine its causal contributions at the subregional level.

Transcranial magnetic stimulation (TMS), a powerful tool that can effectively modulate neural activity of certain cortical regions,^28^ has been employed to probe the causal roles of specific brain regions in vocal motor control. A growing body of research has shown that applying continuous theta burst stimulation (cTBS)^29^ over the left dorsolateral prefrontal cortex (DLPFC), supplementary motor area (SMA), right posterior STG, bilateral middle temporal gyrus (MTG), bilateral supramarginal gyrus (SMG), and right cerebellum can elicit region-specific changes in vocal responses to pitch perturbations, accompanied by modulations of corresponding N1 and P2 responses.^30^^,^^31^^,^^32^^,^^33^^,^^34^^,^^35^^,^^36^ Despite these advances, traditional TMS protocols primarily characterize stimulation effects through changes in behavioral and/or neural responses. While such effects indicate that the targeted region contributes to task performance, they do not reveal how stimulation-induced perturbations propagate through interconnected neural networks, nor do they characterize network-level interactions among distributed regions. Complementing TMS with network-level analyses may therefore provide additional information about how stimulation-related changes are represented across interconnected cortical regions during vocal motor control.

To this end, the present study proposed an integrated approach combining neuronavigated cTBS with source-space network modeling within a causal inference analysis (CIA) framework (see Figure 1) to investigate the role of the left IFG in vocal motor control via auditory feedback at the subregional level. The CIA framework integrated three complementary methods to characterize stimulation-induced network patterns: (1) Bayesian network construction, which models directed probabilistic relationships between regions to estimate condition-dependent dependencies among cortical regions^37^; (2) Granger causality analysis, which quantifies directional temporal predictability between neural signals to characterize model-inferred temporal dependencies^38^; and (3) counterfactual estimation, which provides a statistical approach for comparing observed responses under the actual stimulation condition with estimated responses under alternative stimulation conditions.^39^ Participants produced sustained vocalizations while hearing their voice unexpectedly pitch-shifted in auditory feedback following active or sham cTBS over the left aIFG or pIFG (see Figure 2). Vocal and ERP (N1 and P2) responses to pitch perturbations were measured to assess the effects of stimulation, with N1 typically reflecting early detection of auditory feedback perturbations and P2 more closely reflecting later-stage processes related to auditory-motor integration.^30^^,^^31^ This integrated approach combined behavioral and neurophysiological measures with source-space modeling of stimulation-related network patterns, allowing us to examine the role of the left IFG in vocal motor control and characterize stimulation-related patterns of distributed network organization.

**Figure 1:**
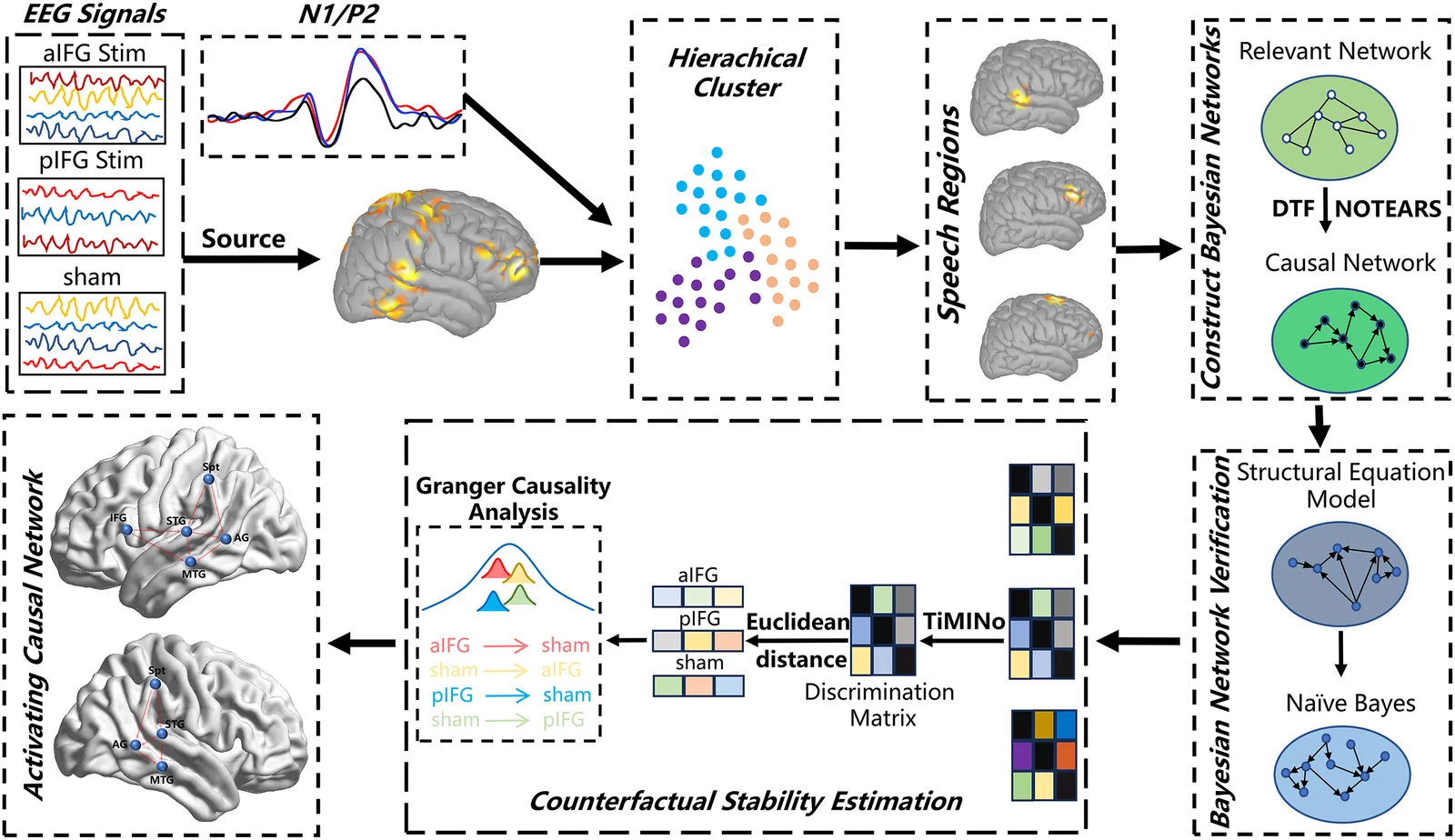
Overview of the CIA framework used to characterize directed functional connectivity patterns following cTBS over the left aIFG and pIFG Source-localized ERP signals were parcellated into cortical regions and used for Bayesian network construction. The NOTEARS algorithm was applied to optimize sparse acyclic network structures. SEM, classifier-based discrimination, counterfactual estimation, and robustness analyses were used as internal model-assessment and sensitivity procedures. Granger causality analysis was used to characterize model-inferred directed dependencies between regions. aIFG: anterior inferior frontal gyrus; pIFG: posterior inferior frontal gyrus; NOTEARS: non-combinatorial optimization via trace exponential and augmented lagRangian for structure learning; SEM: structural equation modeling; BDeu: Bayesian dirichlet equivalent uniform; DTF: directed transfer function; TiMINo: Time series models with independent noise.

**Figure 2:**
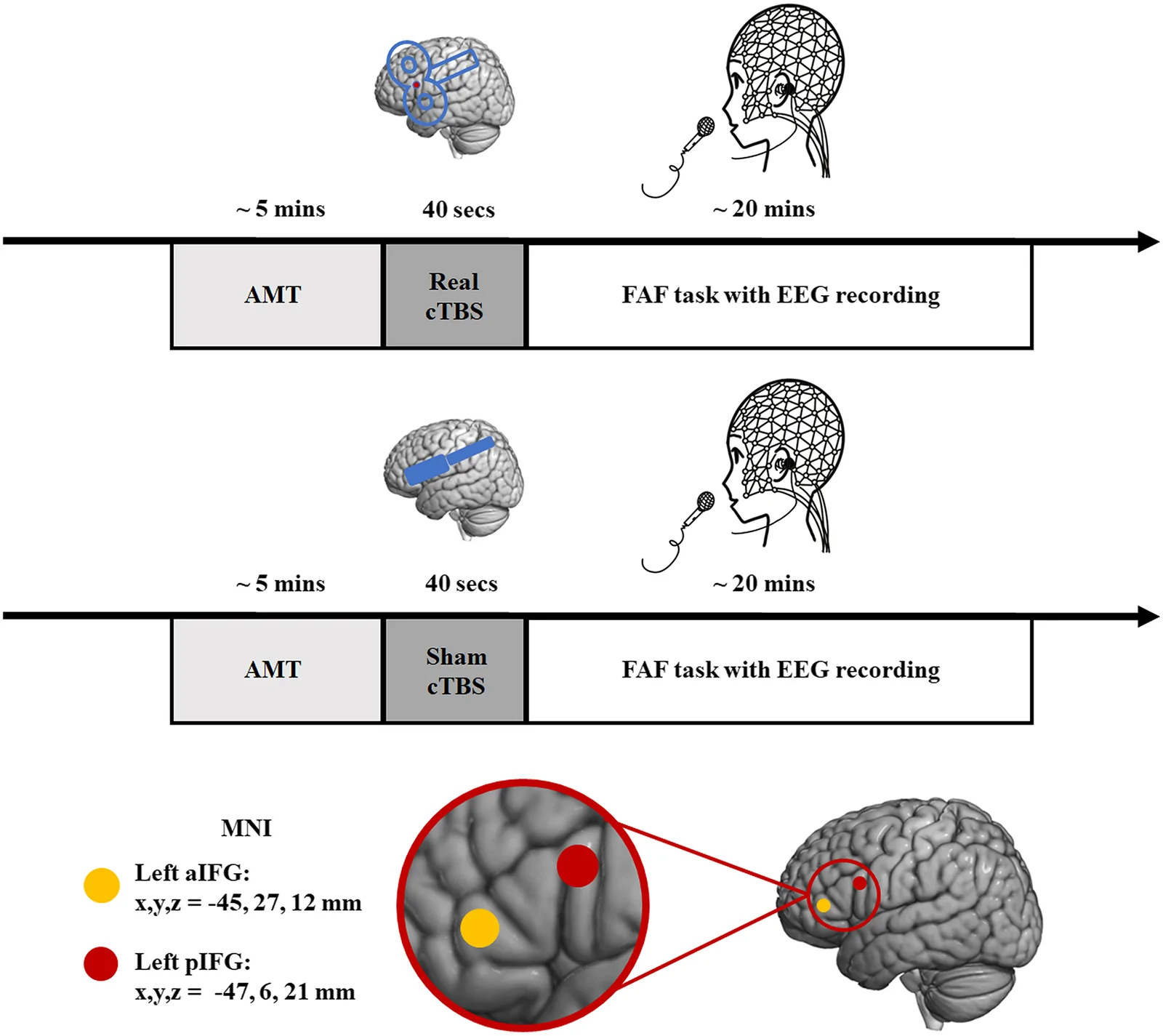
Illustration of the experimental setup First, the active motor threshold (AMT) was measured to determine the intensity of cTBS on an individual basis. The participants then received active (top) or sham (middle) cTBS, administered for 40 seconds for a total of 600 pulses, targeting the left aIFG or pIFG with MRI-guided neuronavigation (bottom). Immediately following the stimulation, participants were instructed to perform a vocal production experiment based on the frequency-altered feedback (FAF) paradigm, during which they produced sustained vowel phonations while hearing their voice unexpectedly shifted in pitch by +200 or −200 cents. The voice and electroencephalographic (EEG) signals were recorded simultaneously for the assessment of behavioral and ERP responses across different stimulation conditions. aIFG: anterior inferior frontal gyrus; pIFG: posterior inferior frontal gyrus.

## Results

### Behavioral findings

Figures 3A and 3B show the grand-averaged voice *f*_o_ contours in response to pitch perturbations as a function of perturbation direction across stimulation sessions. A two-way RM-ANOVA conducted on the peak magnitudes of vocal responses revealed a significant main effect of stimulation session (F[2, 40] = 24.766, *p* < 0.001, $\eta_{p}^{2}$ = 0.533) (see Figure 3C), where sham stimulation led to larger vocal responses to pitch perturbations than cTBS over the left aIFG (*p* = 0.001) and pIFG (*p* < 0.001). No significant difference, however, was found between cTBS over the left aIFG and left pIFG (*p* = 0.263). In addition, the main effect of perturbation direction (F[1, 20] = 0.991, *p* = 0.331) and its interaction with stimulation session (F[2, 40] = 1.208, *p* = 0.310) did not reach significance. Figures 3E and 3F show the directional effects of cTBS over the left aIFG and pIFG on vocal responses relative to sham stimulation at the individual level, respectively. Specifically, in response to both +200 and −200 cents perturbations, a decrease in the magnitude was observed in 36 out of 42 vocal responses following cTBS over the left aIFG. Similarly, cTBS over the left pIFG resulted in a decrease in 40 out of 42 vocal responses.

**Figure 3:**
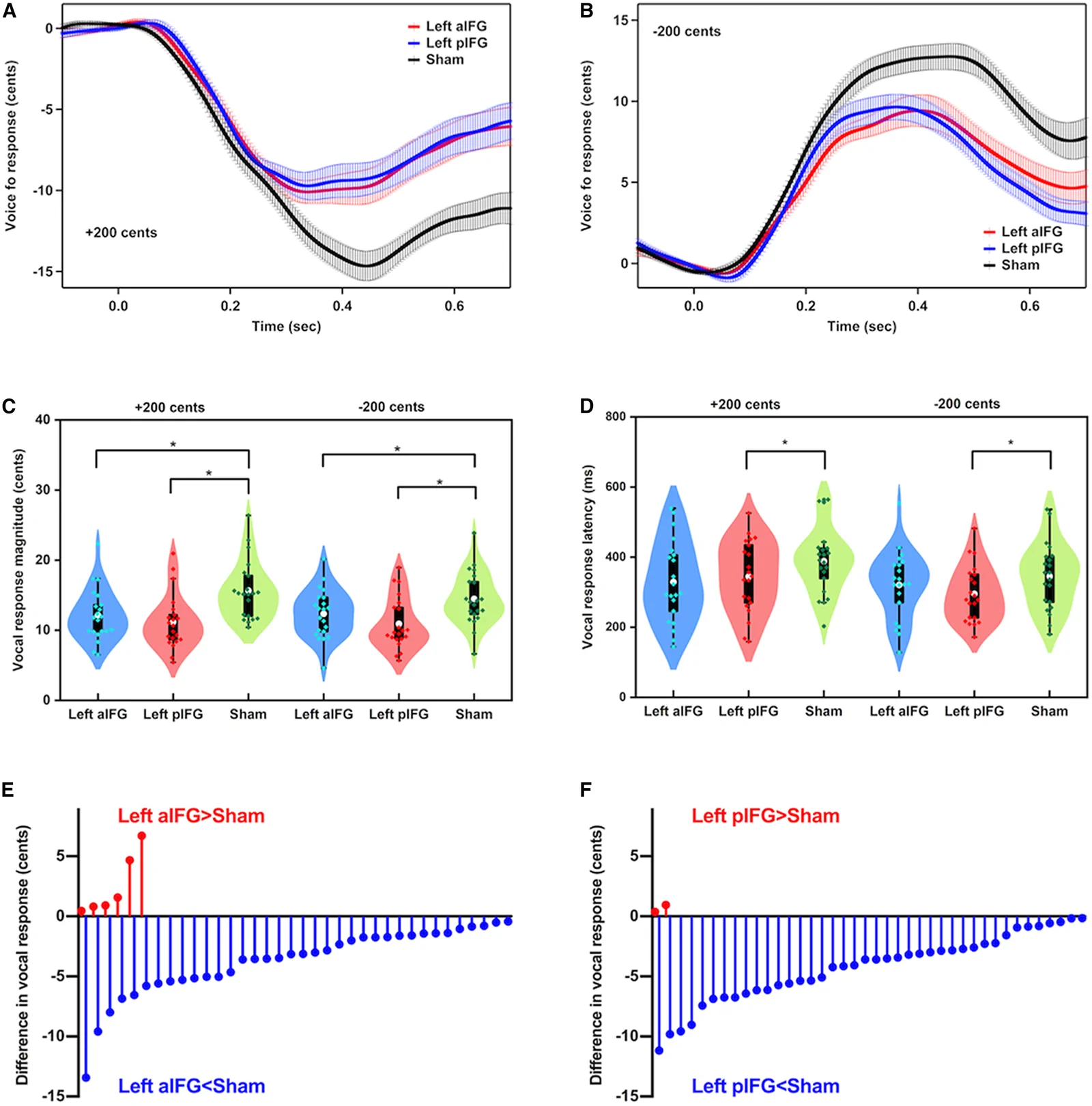
Effects of left aIFG and pIFG cTBS on vocal responses to pitch perturbations (A and B) Grand-averaged voice *f*_o_ responses to pitch perturbations of +200 and −200 cents following cTBS over the left aIFG (red) and pIFG (blue) as well as sham stimulation (black). Vertical bars across the voice *f*_o_ contours represent the standard errors of the mean vocal responses. (C and D) Violin plots of the peak magnitudes and latencies of vocal responses to pitch perturbations of +200 and −200 cents following cTBS over the left aIFG (blue) and pIFG (red) as well as sham stimulation (green). The white dots and black boxplots represent the medians and ranges from the first to third quartiles of the datasets. The blue, red, and green dots represent the individual vocal responses to pitch perturbations. The asterisks indicate significant differences across the conditions. (E and F) Individual variability in the direction of behavioral effects of cTBS on vocal responses to pitch perturbations, as reflected by differences in the peak magnitudes of vocal responses between cTBS over the left aIFG or pIFG and sham stimulation. Left aIFG <Sham and Left pIFG <Sham indicate reduced vocal responses following two active stimulations relative to sham stimulation, while Left aIFG >Sham and Left pIFG >Sham indicate the opposite effects. aIFG: anterior inferior frontal gyrus; pIFG: posterior inferior frontal gyrus.

Regarding the peak time of vocal response, there was a significant main effect of stimulation session (F[2, 40] = 3.363, *p* = 0.045, $\eta_{p}^{2}$ = 0.144), indicating that longer time was required to reach the peak magnitude of vocal response for sham stimulation than for cTBS over the left pIFG (*p* = 0.038) (see Figure 3D). No significant difference was found between cTBS over the left aIFG and sham stimulation (*p* = 0.216) or between cTBS over the left aIFG and pIFG (*p* = 1.000). In addition, the main effect of perturbation direction (F[1, 20] = 4.041, *p* = 0.058) and its interaction with stimulation session (F[2, 40] = 0.523, *p* = 0.597) were not statistically significant.

### ERP findings

Figures 4A and 4B show the grand-averaged ERPs to pitch perturbations at the frontal, fronto-central, and central electrode sites, as well as the topographic distributions of the N1 and P2 responses across the conditions. A three-way RM-ANOVA conducted on the N1 amplitude revealed a significant main effect of perturbation direction (F[1, 20] = 7.289, *p* = 0.014, $\eta_{p}^{2}$ = 0.267), indicating smaller N1 responses to upward perturbations than to downward perturbations (see Figure 4C). There was a significant main effect of electrode site (F[2, 40] = 4.083, *p* = 0.044, $\eta_{p}^{2}$ = 0.185), as well as a significant interaction between electrode site and perturbation direction (F[2, 40] = 8.233, *p* = 0.004, $\eta_{p}^{2}$ = 0.292). Follow-up analyses revealed a significant main effect of electrode site (F[2, 40] = 6.902, *p* = 0.006, $\eta_{p}^{2}$ = 0.257) for upward perturbations, where N1 amplitudes were smaller at frontal electrodes than at fronto-central electrodes (*p* = 0.003). No such significant difference, however, was observed for downward perturbations (F[2, 40] = 1.252, *p* = 0.288). In addition, there was no significant main effect of stimulation session (F[2, 40] = 2.257, *p* = 0.118), nor any significant interactions among the three factors (all *p* > 0.3).

**Figure 4:**
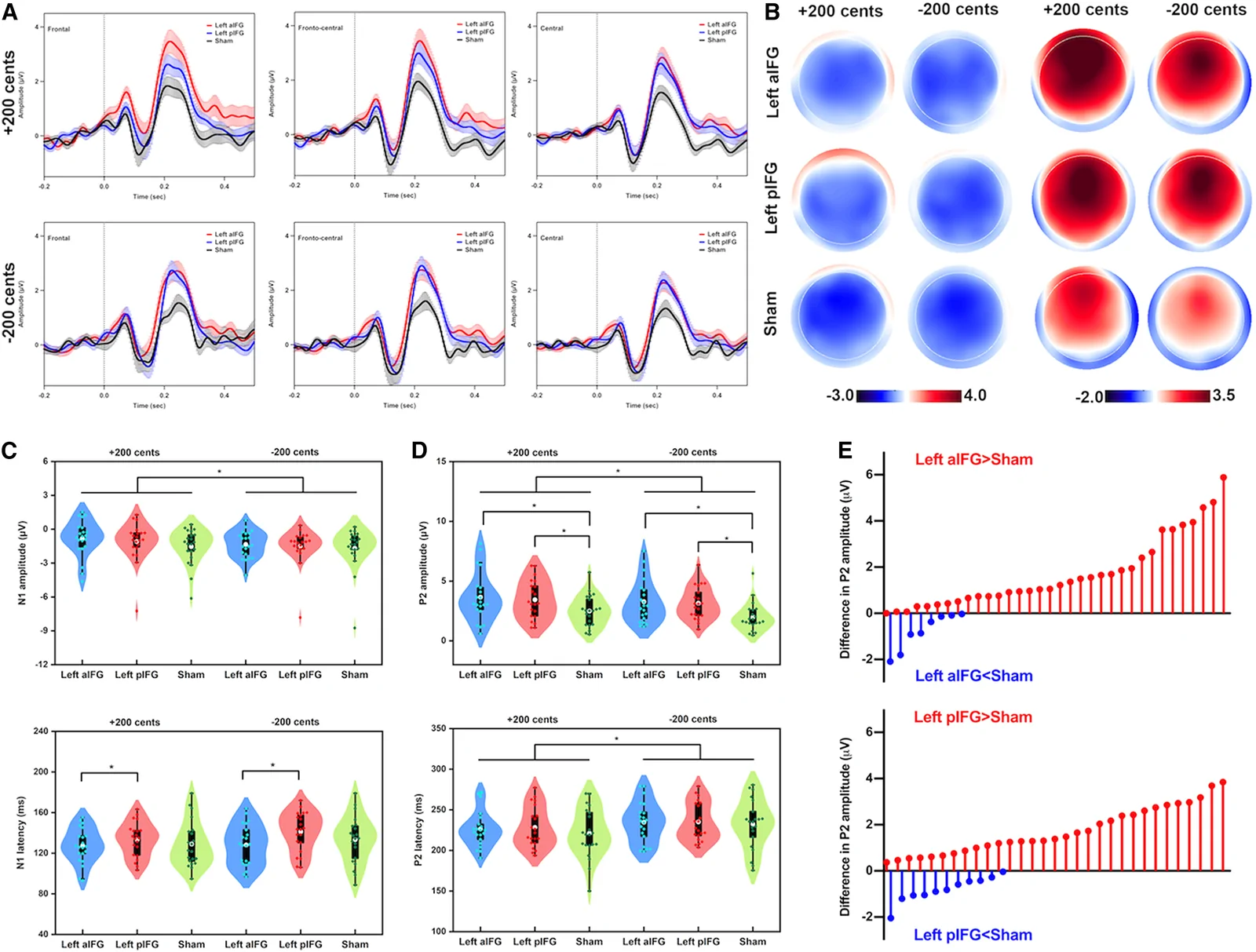
Effects of left aIFG and pIFG cTBS on ERP responses to pitch perturbations (A and B) Grand-averaged ERP waveforms and topographic distributions of the N1 and P2 responses to pitch perturbations of +200 and −200 cents following cTBS over the left aIFG and pIFG as well as sham stimulation. Vertical bars across the ERP waveforms represent the standard errors of the mean ERP responses. (C and D) Violin plots of the amplitudes and latencies of the N1 and P2 responses to pitch perturbations of +200 and −200 cents following cTBS over the left aIFG (blue) and pIFG (red) as well as sham stimulation (green). The white dots and black boxplots represent the medians and ranges from the first to third quartiles of the datasets. The blue, red, and green dots represent the individual ERP responses to pitch perturbations. The asterisks indicate significant differences across the conditions. (E) Individual variability in the direction of ERP effects of cTBS on vocal pitch regulation, as reflected by differences in the amplitudes of the P2 responses between cTBS over the left aIFG or pIFG and sham stimulation. Left aIFG >Sham and Left pIFG >Sham indicate enhanced P2 responses following the two active stimulations relative to sham stimulation, while Left aIFG <Sham and Left pIFG <Sham indicate the opposite effects. aIFG: anterior inferior frontal gyrus; pIFG: posterior inferior frontal gyrus.

Regarding N1 latency, there was a significant main effect of stimulation session (F[2, 40] = 3.576, *p* = 0.037, $\eta_{p}^{2}$ = 0.152), indicating faster N1 responses following cTBS over the left aIFG compared to cTBS over the left pIFG (*p* = 0.013) (see Figure 4C). However, no significant differences were found between sham stimulation and cTBS over the left aIFG (*p* = 1.000) or pIFG (*p* = 0.381). In addition, there was no significant main effect of electrode site (F[2, 40] = 1.127, *p* = 0.334) or perturbation direction (F[1, 20] = 2.899, *p* = 0.104), nor any significant interactions among any of the three factors (all *p* > 0.2).

A three-way RM-ANOVA conducted on the P2 amplitude showed a significant main effect of stimulation session (F[2, 40] = 7.700, *p* = 0.001, $\eta_{p}^{2}$ = 0.278). Compared to sham stimulation, P2 amplitudes were significantly increased following cTBS over both the left aIFG (*p* = 0.004) and pIFG (*p* = 0.003), whereas no significant differences were found between cTBS over the left aIFG and left pIFG (*p* = 1.000) (see Figure 4D). There was also a significant main effect of electrode site (F[2, 40] = 24.132, *p* < 0.001, $\eta_{p}^{2}$ = 0.547), indicating smaller P2 amplitudes at central electrodes compared to frontal (*p* < 0.001) and fronto-central electrodes (*p* < 0.001). A significant main effect of perturbation direction (F[1, 20] = 8.742, *p* = 0.008, $\eta_{p}^{2}$ = 0.304) was also found, showing larger P2 responses to upward perturbations than to downward perturbations. In addition, no significant interactions were found among any of the three factors (all *p* > 0.2). Figure 4E shows the individual variability in the directional effects of cTBS over the left aIFG and pIFG on the P2 responses relative to sham stimulation. In response to both +200 and −200 cents perturbations, 34 out of 42 P2 responses showed enhancement following cTBS over the left aIFG. Similarly, cTBS over the left pIFG resulted in an increase in 31 out of 42 P2 responses.

Regarding P2 latency, there was a significant main effect of perturbation direction (F[1, 20] = 8.233, *p* = 0.009, $\eta_{p}^{2}$ = 0.292), indicating faster P2 responses to upward perturbations than to downward perturbations (see Figure 4D). However, the main effects of stimulation session (F[2, 40] = 0.326, *p* = 0.724) and electrode site (F[2, 40] = 2.037, *p* = 0.162) were not significant, nor were any significant interactions found among the three factors (all *p* > 0.5).

Figure 5 shows the global dissimilarity (DISS) results for the aIFG-sham, pIFG-sham, and aIFG-pIFG contrasts within the N1 and P2 time windows as a function of perturbation direction. The upper images show the spatial distribution of normalized topographic differences for the N1 and P2 components under upward and downward pitch perturbations, and the lower images show the corresponding group-level DISS comparisons for all three contrasts. For the N1 time window, significant main effects of contrast were observed for both upward (F[2, 40] = 15.675, *p* < 0.001, $\eta_{p}^{2}$ = 0.439) and downward (F[2, 40] = 11.198, *p* < 0.001, $\eta_{p}^{2}$ = 0.359) perturbations. Bonferroni-corrected comparisons showed that DISS values for the aIFG-pIFG contrast were significantly smaller than those for both the aIFG-sham and pIFG-sham contrasts under upward and downward perturbations (all *p* < 0.001). In contrast, DISS values did not differ significantly between the aIFG-sham and pIFG-sham contrasts under either upward (*p* = 0.204) or downward (*p* = 0.148) perturbations.

**Figure 5:**
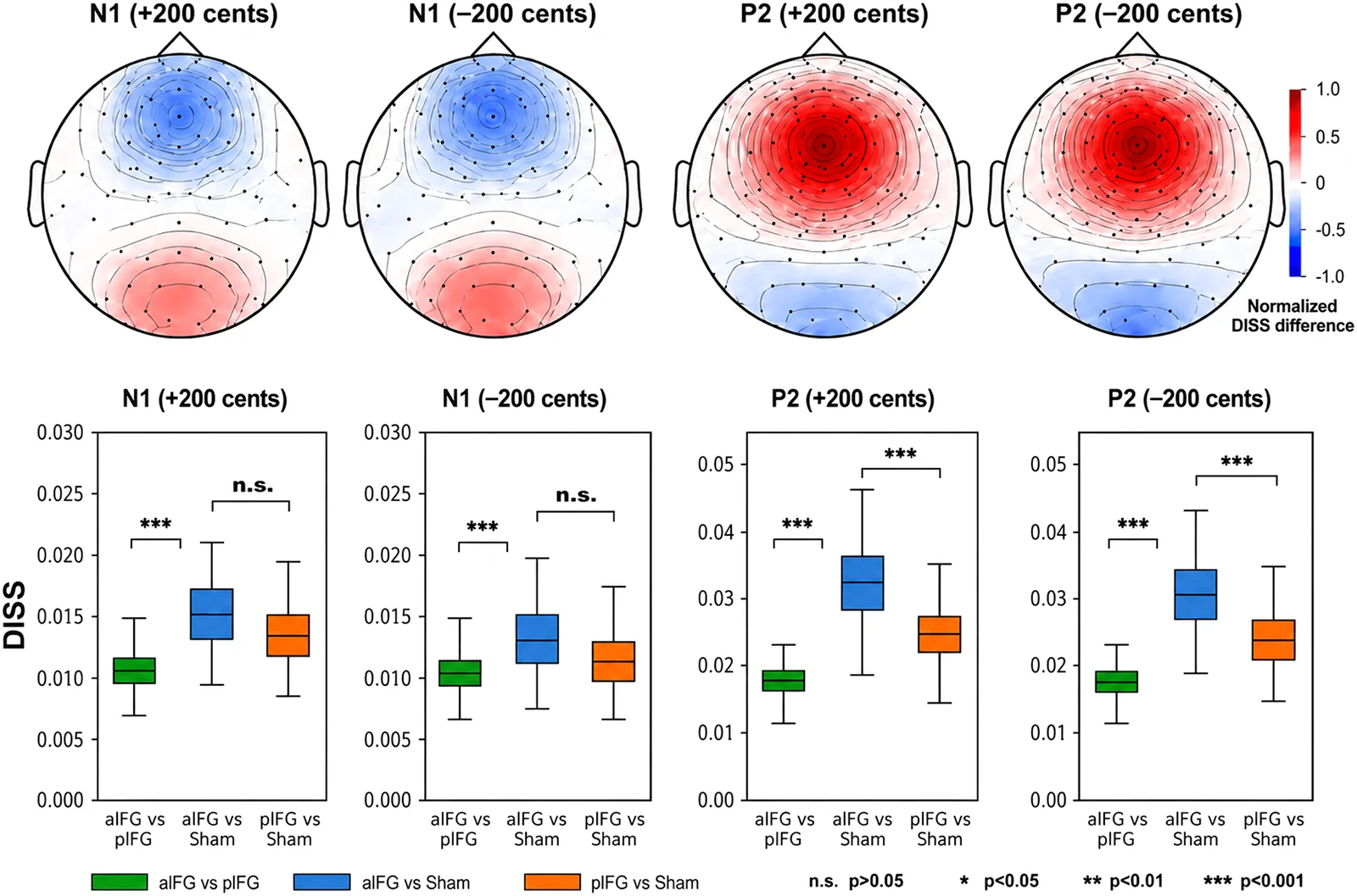
DISS analysis of stimulation-related ERP topographic changes The upper images show the spatial distribution of normalized topographic differences between left aIFG and left pIFG conditions for the N1 and P2 components under upward (+200 cents) and downward (−200 cents) pitch perturbations. Images on the lower part show group-level DISS values for the aIFG-pIFG (green), aIFG-sham (blue), and pIFG-sham (orange) contrasts. Boxes indicate the interquartile range, horizontal lines indicate medians, and whiskers indicate the minimum and maximum values. ∗∗∗ above the aIFG-pIFG boxes indicates significantly smaller DISS than both aIFG-sham and pIFG-sham contrasts (*p* < 0.001), whereas ∗∗∗ above a bracket indicates a significant difference between the two bracketed contrasts. aIFG: anterior inferior frontal gyrus; pIFG: posterior inferior frontal gyrus; DISS: global dissimilarity; n.s.: not significant. ∗∗∗ *p* < 0.001 after Bonferroni correction.

For the P2 time window, significant main effects of contrast were observed for both upward (F[2, 40] = 177.186, *p* < 0.001, $\eta_{p}^{2}$ = 0.898) and downward (F[2, 40] = 181.103, *p* < 0.001, $\eta_{p}^{2}$ = 0.900) perturbations. Bonferroni-corrected comparisons showed that DISS values for the aIFG-pIFG contrast were significantly smaller than those for both the aIFG-sham and pIFG-sham contrasts under both perturbation directions (all *p* < 0.001). In addition, DISS values were significantly larger for the aIFG-sham contrast than for the pIFG-sham contrast under both upward and downward perturbations (all *p* < 0.001).

### Causal inference analysis

Given significant effect of stimulation session on P2 amplitude, P2-window source-space ERP data were submitted to the CIA model to characterize network patterns of directed functional connectivity associated with cTBS over the left aIFG and pIFG. In this model assessment, a non-significant *p* value indicates that the observed connectivity pattern did not significantly deviate from the estimated directed network structure.

When comparing cTBS over the left aIFG with sham stimulation, the CIA revealed no significant deviation between the observed connectivity pattern and the estimated network structure (F[4, 428] = 0.8467, *p* = 0.496). Granger causality analysis identified directed dependencies involving regions associated with high-order auditory processing (see Figure 6), including the STG, IFG, angular gyrus (AG), Sylvian parietal-temporal area (Spt), and MTG. In the left hemisphere, directed dependencies were identified from the IFG to both STG and MTG. Such directed dependencies were also observed from the STG to AG and MTG, from the Spt to STG and AG, and from the AG to MTG. In the right hemisphere, directed dependencies were observed from the Spt to the STG and AG, from the STG to the AG and MTG, and from the AG to the MTG.

**Figure 6:**
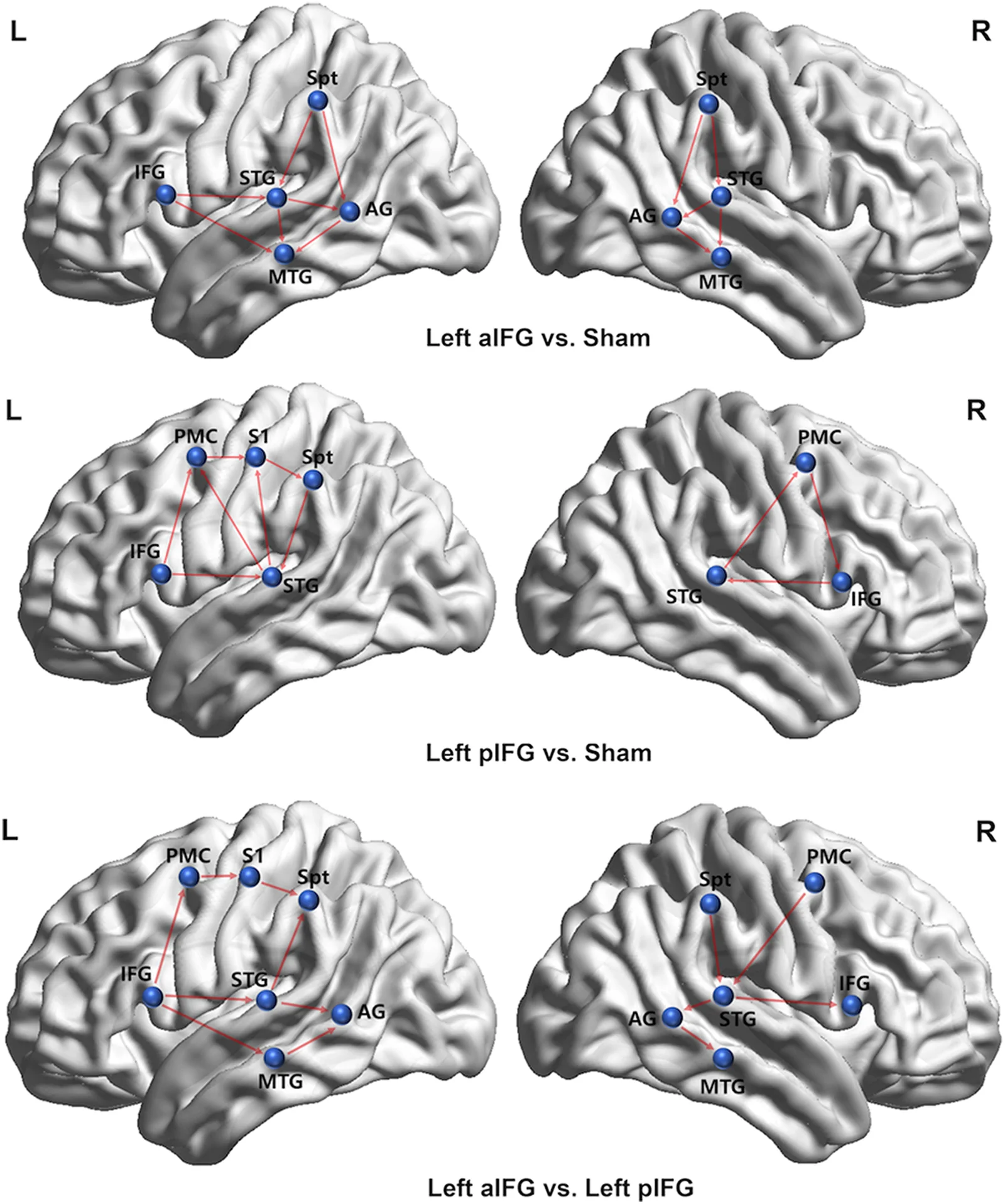
Model-inferred directional source-space network patterns for the three stimulation contrasts derived from Granger causality analysis within the CIA framework The upper row shows the aIFG-sham contrast, involving the left IFG and bilateral STG, MTG, AG, and Spt. The middle row shows the pIFG-sham contrast, involving the bilateral IFG, STG, and PMC, together with the left S1 and Spt. The lower row shows the aIFG-pIFG contrast, involving the bilateral IFG, STG, MTG, AG, PMC, and Spt, together with the left S1. Blue spheres indicate regional network nodes, and red arrows indicate model-inferred directional dependencies, with arrowheads denoting direction. L and R indicate the left and right hemispheres, respectively. IFG: inferior frontal gyrus; aIFG: anterior inferior frontal gyrus; pIFG: posterior inferior frontal gyrus; STG: superior temporal gyrus; MTG: middle temporal gyrus; AG: angular gyrus; PMC: premotor cortex; S1: primary somatosensory cortex; Spt: Sylvian parietal-temporal area; CIA: causal inference analysis.

When comparing cTBS over the left pIFG with sham stimulation, the CIA also revealed no significant deviation between the estimated network structure and the observed connectivity pattern (F[4, 428] = 0.980, *p* = 0.417). Granger causality analysis identified directed dependencies involving regions associated with sensorimotor transformation, including the STG, IFG, PMC, primary somatosensory cortex (S1), and Spt (see Figure 6). In the left hemisphere, directed dependencies were identified from the IFG to PMC and STG. Additional directed dependencies were also observed from the STG to the PMC, S1, and Spt, from the PMC to S1, from the S1 to Spt, and from the Spt to STG. In the right hemisphere, directed dependencies were identified from the STG to the PMC, from the PMC to the IFG, and from the IFG to the STG.

When comparing cTBS over the left aIFG with cTBS over the left pIFG, the CIA revealed no significant deviation between the estimated network structure and the observed connectivity pattern (F[4, 428] = 0.913, *p* = 0.456). Granger causality analysis identified directed network organization involving the IFG, STG, MTG, AG, PMC, S1, and Spt (see Figure 6). In the left hemisphere, directed dependencies were identified from the IFG to the PMC, STG, and MTG, from the PMC to S1, from S1 to Spt, from the STG to Spt and AG, and from the MTG to AG. In the right hemisphere, directed dependencies were identified from the Spt and PMC to the STG, from the STG to the IFG and AG, and from the AG to the MTG. Thus, the aIFG-pIFG comparison revealed a distributed network configuration involving regions associated with both auditory processing and sensorimotor integration.

Cross-inverse sensitivity analyses showed substantial convergence across MNE, wMNE, sLORETA, and LAURA (Figures S1–S3). Pairwise source-map correlations ranged from 0.69 to 0.82 for aIFG-pIFG, 0.72 to 0.86 for aIFG-sham, and 0.70 to 0.84 for pIFG-sham. The corresponding node-overlap Jaccard indices were 0.74, 0.78, and 0.81, and directed-edge overlap indices were 0.61, 0.65, and 0.69, respectively. Participant-level and bootstrap analyses further indicated the stability of several principal directed dependencies (Table S3). In the aIFG-sham network, directed dependencies from the left IFG to the left STG and from the left IFG to the left MTG were recovered in 18/21 (85.7%) and 17/21 (81.0%) participants, with corresponding bootstrap occurrence rates of 87.4% and 83.1%, respectively. In the pIFG-sham network, directed dependencies from the left IFG to the left PMC and from the left IFG to the left STG were recovered in 18/21 (85.7%) and 17/21 (81.0%) participants, with corresponding bootstrap occurrence rates of 86.6% and 84.2%, respectively. For the direct aIFG-pIFG comparison, principal directed edges showed participant-level recurrence ranging from 66.7% to 76.2% and bootstrap occurrence ranging from 72.3% to 79.6%. Together, these analyses showed substantial convergence of the principal regional and network-level patterns across inverse solutions and stability across participant-level and bootstrap assessments.

## Discussion

By integrating neuronavigated cTBS with a novel CIA framework, the present study examined the role of the left IFG in vocal motor control at both behavioral and neural levels. Our results showed that cTBS over both the left aIFG and pIFG reduced vocal responses to pitch perturbations and enhanced P2 responses compared to sham stimulation, with no significant differences in these neurobehavioral responses between the two active stimulation conditions. Notably, the DISS analysis showed that the aIFG-sham contrast yielded larger topographic differences than the pIFG-sham contrast in the P2 time window. Furthermore, our CIA model identified different patterns of directed network organization associated with the two stimulation targets. Specifically, left aIFG stimulation involved the IFG, STG, MTG, AG, and Spt, whereas left pIFG stimulation involved the IFG, STG, PMC, S1, and Spt. The direct aIFG-pIFG comparison further revealed differences in distributed network organization involving the IFG, STG, MTG, AG, PMC, S1, and Spt. These findings not only provide evidence supporting the causal involvement of the left IFG in vocal motor control, but also suggest that stimulation of its anterior and posterior regions may be associated with different patterns of topographic and network organization.

Despite the established role of the left IFG in speech planning and execution,^11^^,^^16^^,^^17^^,^^18^ its precise role in auditory feedback control of vocal production remains incompletely understood. The DIVA model^14^ and several neuroimaging studies^7^^,^^8^^,^^10^^,^^11^ have emphasized the involvement of the right IFG in processing auditory or somatosensory feedback errors during speech production, whereas other studies have implicated the left IFG in this feedback-based control process.^19^^,^^26^^,^^27^ Recent research has begun to address this issue using non-invasive brain stimulation approaches, showing that HD-tACS over the left IFG at 6 or 70 Hz led to enhanced vocal and cortical P2 responses to pitch perturbations.^20^ Although these findings support a role of the left IFG in vocal motor control, they do not determine whether left IFG subregions contribute similarly or differently to this process. To address this question, the present study employed neuronavigated cTBS to stimulate the anterior and posterior left IFG separately. Our findings showed that cTBS over either the left aIFG or left pIFG led to reduced vocal responses to pitch perturbations accompanied by enhanced P2 amplitudes, supporting a causal contribution of both anterior and posterior left IFG subregions to vocal motor control.

Compared to sham stimulation, cTBS over both the left aIFG and pIFG reduced peak magnitudes of vocal responses, and cTBS over the left pIFG also shortened peak times. These patterns align with previous studies on vocal feedback control that applied cTBS over the left SMA,^31^ left/right SMG,^33^^,^^36^ right posterior STG,^34^ and left/right/bilateral posterior MTG.^35^ Notably, enhanced magnitudes and/or delayed timings of vocal responses have been observed in neurological diseases such as Parkinson’s disease (PD),^27^^,^^40^^,^^41^ Alzheimer’s disease (AD),^42^ stroke,^43^ and spinocerebellar ataxia (SCA),^44^^,^^45^ which have been thought to reflect impaired or slowed sensorimotor control of speech production.^30^^,^^42^ In contrast, attenuated and/or accelerated vocal responses have been reported in individuals undergoing cognitive or musical training^8^^,^^46^ and in PD patients receiving voice treatment.^47^^,^^48^ In this context, our findings of reduced/accelerated vocal responses following cTBS over left IFG subregions may reflect reduced reliance on auditory feedback error signals during vocal motor control.^8^^,^^30^

Furthermore, although cTBS is typically considered to reduce cortical excitability at the targeted site,^29^ cTBS over either the left aIFG or left pIFG led to enhanced cortical P2 responses to vocal pitch perturbations in the present study. Similar P2 enhancements were also observed following cTBS over the right STG^34^ and left/right/bilateral MTG.^35^ The P2 component has been implicated in later-stage auditory-motor integration for vocal feedback control.^8^^,^^30^^,^^49^ This interpretation is supported by several findings that have shown significant correlations between larger P2 amplitudes and smaller vocal responses.^8^^,^^31^^,^^32^^,^^34^^,^^48^ By contrast, cTBS over the two left IFG subregions did not significantly modulate the N1 amplitude, which is typically associated with early detection of auditory feedback errors.^50^^,^^51^ This pattern suggests that stimulation of the left IFG may primarily modulate later-stage auditory-motor integration that guides corrective vocal responses, rather than altering the early detection of pitch perturbations.

One plausible explanation for P2 enhancement following cTBS over the left aIFG or pIFG lies in stimulation-related differences in distributed network organization mediated by cortico-cortical interactions.^52^ Concurrent TMS-fMRI studies have shown that cTBS over a targeted area can lead to increased activity in the contralateral area,^25^^,^^53^ suggesting that disrupting neural activity in one hemisphere may be counterbalanced by increased activity in the contralateral hemisphere. Furthermore, cTBS has been shown to inhibit activity at the stimulation site while enhancing activity in remote but functionally interconnected brain regions.^54^^,^^55^ In this context, cTBS over the left IFG may trigger compensatory or additional recruitment of distributed cortical networks that support vocal feedback control, resulting in increased cortical activity in the P2 time window. This interpretation is consistent with our CIA findings, which identified distributed functional network patterns involving frontal, temporal, and parietal regions following cTBS over the left aIFG and pIFG. Further support comes from studies showing that low-frequency rTMS over the right STG in PD patients enhanced functional connectivity with articulatory-related regions.^56^^,^^57^

Although vocal and ERP measures did not differ significantly between cTBS over the left aIFG and left pIFG, the DISS analysis revealed a component-specific difference in the topographic configuration of the ERP effects induced by stimulation of the two left IFG subregions. DISS provides a complementary measure of whether experimental conditions differ in response topography independent of global response strength.^58^ In the N1 time window, DISS values did not differ between the aIFG-sham and pIFG-sham contrasts, suggesting that cTBS over the left aIFG and left pIFG produced comparable scalp-field configurations during the early stage of feedback-error detection. By contrast, significantly larger DISS values were observed for the aIFG-sham contrast than for the pIFG-sham contrast in the P2 time window, suggesting that left aIFG stimulation produced a greater alteration in the spatial configuration of P2-related cortical activity relative to sham stimulation than did left pIFG stimulation. In addition, across both the N1 and P2 time windows, DISS values were smaller for the aIFG-pIFG contrast than for either contrast with sham, indicating greater similarity in scalp-field configuration between the two active stimulations than between either active condition and sham stimulation. Taken together, these findings suggest a broadly similar scalp-level organization following stimulation of the anterior and posterior left IFG, with differential topographic modulation emerging during the later P2 stage.

The CIA results further suggested different patterns in source-space network organization associated with stimulation of the left aIFG and pIFG. Specifically, cTBS over the left aIFG was associated with changes in directed functional connectivity within a distributed network involving the STG, IFG, AG, Spt, and MTG, regions that have been implicated in higher-order auditory and phonological processing. In contrast, cTBS over the left pIFG was associated with model-inferred directional dependencies within a distributed network including the STG, IFG, PMC, S1, and Spt, regions that have been implicated in sensorimotor transformation and articulatory control during speech production. Note that the direct aIFG-pIFG comparison revealed changes in directional dependencies within a distributed network involving the IFG, STG, MTG, AG, PMC, S1, and Spt, despite the absence of significant neurobehavioral differences between these two active stimulations. These findings extend dual-stream models of speech processing^2^^,^^15^^,^^59^ by suggesting that stimulation of the anterior and posterior parts of the left IFG may differentially influence the organization of auditory and sensorimotor networks during vocal motor control. This pattern also lends further support to previous neuroimaging studies linking both dorsal and ventral streams to speech motor control.^6^^,^^7^^,^^8^^,^^9^^,^^10^^,^^11^^,^^34^

Moreover, our CIA results further characterized the model-inferred directed dependencies within the functional networks associated with stimulation of each left IFG subregion. Following left aIFG stimulation, the CIA identified directed dependencies from the IFG to the STG and MTG in the left hemisphere, consistent with previously reported left IFG-STG connectivity during vocal pitch regulation.^19^ Additional directed dependencies from the STG to both the AG and MTG, and from the Spt to the STG and AG, suggest the involvement of these temporal and parietal regions in the processing of auditory feedback^7^^,^^9^^,^^10^ and sensorimotor transformation^2^^,^^60^ during speech production. This interpretation is consistent with lesion evidence showing that damage to the orbital part of the left IFG was associated with reduced vocal responses to pitch perturbations during the P2 (150–250 ms) window.^26^ Together, these findings extend the traditional understanding of the left aIFG beyond its established role in syntactic and semantic processing,^61^^,^^62^ suggesting the involvement of this region in top-down modulation of auditory feedback within distributed speech motor networks.

Within the network associated with left pIFG stimulation, the CIA identified directed dependencies from the IFG to both the PMC and STG in the left hemisphere, consistent with previous evidence linking the left pIFG to the integration of auditory feedback with corrective motor commands.^19^ Additional directed dependencies from the STG to the PMC, S1, and Spt, from the PMC to S1, and from S1 to Spt suggest coordinated involvement of auditory and sensorimotor regions in transforming sensory information into motor control. These directed dependencies align with empirical and theoretical studies highlighting the role of the dorsal-stream regions in auditory-motor transformation for speech control.^2^^,^^6^^,^^14^ Similar network features were also observed in the right hemisphere, in line with previous research showing the bilateral coordination of sensorimotor networks in auditory-vocal integration.^7^ Traditionally, the left pIFG has been implicated in speech production,^15^ particularly in the DIVA model as a central component of the speech sound map for planning and executing normal speech.^14^ Our CIA results extend this perspective by suggesting that the left pIFG may also be involved in the online monitoring and correction of vocal output through interactions with sensorimotor regions such as the PMC and S1 to translate feedback error signals into targeted motor adjustments. This expanded interpretation supports a distributed framework in which the left pIFG contributes not only to feedforward speech planning but also to feedback control of vocal output.

The aIFG-pIFG comparison further characterized the network-level distinction between stimulation of the two IFG subregions. The differential network involved directed dependencies linking frontal regions with temporal, temporoparietal, and sensorimotor regions, indicating that the distinction extended across multiple components of the auditory-vocal network. This distributed pattern is consistent with the connectional organization of the left IFG. Connectivity-based parcellation has demonstrated distinct connectivity profiles across posterior and anterior Broca’s regions,^63^ while intracranial cortico-cortical evoked potentials have revealed an anterior-posterior connectivity gradient, with posterior left IFG preferentially connected to posterior temporal and supramarginal regions and anterior left IFG to anterior temporal and angular regions.^64^ These anatomical and electrophysiological findings provide a plausible basis for the different network configurations associated with left aIFG and left pIFG stimulation, suggesting that their distinction may arise from differential interactions with distributed temporal-parietal and sensorimotor systems.

Importantly, CIA-derived differences in network organization emerged despite the absence of significant aIFG-pIFG differences in vocal compensation or N1/P2 amplitudes. This pattern can be understood in terms of the different levels of analysis captured by these measures: vocal and ROI-based ERP peak measures quantify the magnitude and timing of task-evoked responses, whereas the CIA framework characterizes model-inferred multiregional dependencies among reconstructed regional source signals. By integrating individualized source reconstruction, Bayesian network modeling, counterfactual estimation, and Granger causality analysis, the CIA framework provides information about distributed network reorganization that is not captured by behavioral or ERP measures. The association of the two active conditions with different network configurations but comparable neurobehavioral responses is compatible with the principle of neural degeneracy, whereby different neural configurations can support similar functional outcomes.^65^ In this sense, CIA extends conventional neurobehavioral analyses by identifying different auditory-sensorimotor network configurations following left aIFG and pIFG stimulation, despite no significant differences in vocal compensation or N1/P2 amplitudes.

In summary, the present study showed that cTBS over either the left aIFG or pIFG reduced vocal compensation and enhanced P2 responses to pitch perturbations, with no significant differences between the two active stimulations in these measures. DISS analysis showed greater P2 topographic deviation from sham stimulation following left aIFG than left pIFG stimulation. CIA further identified different model-inferred source-space network configurations associated with the two subregions, and the direct aIFG-pIFG comparison showed that these differences involved distributed auditory and sensorimotor regions. Together, these findings provide causal evidence that the left IFG contributes to auditory-vocal integration and indicate differentiated topographic and network-level effects of stimulating its anterior and posterior regions, highlighting the potential of CIA to complement conventional neurobehavioral analyses by characterizing distributed source-space network organization in vocal feedback control.

### Limitations of the study

Several limitations of the present study should be acknowledged. First, although MRI-guided neuronavigation allowed stimulation over the left aIFG and pIFG, these two subregions are anatomically close and stimulation effects cannot be assumed to be confined exclusively to the intended target. The two active stimulations may produce partially overlapping effects, which could contribute to the absence of significant differences in vocal responses and ERP measures between them. Second, although individualized MRI-based head modeling, BEM forward modeling, and distributed source reconstruction improved the anatomical constraints and stabilized regional estimates in the CIA modeling, these procedures cannot eliminate source leakage, spatial crosstalk, signal mixing, and model dependence. Accordingly, the directed edges derived from the CIA framework should be interpreted as model-inferred directional dependencies among reconstructed regional signals, rather than definitive evidence of physiological causal transmission between cortical regions. Third, the counterfactual estimation, SEM, classifier-based discrimination, and robustness analyses were used as internal model-assessment and sensitivity procedures, rather than independent out-of-sample validation. The CIA findings should therefore be viewed as exploratory network-level evidence that complements the behavioral, ERP, and DISS results. Future studies using complementary modalities such as higher-density EEG/MEG and concurrent TMS-fMRI are needed to explore the proposed network mechanisms more directly.

## Resource availability

### Lead contact

Further information and requests for resources should be directed to and will be fulfilled by the lead contact, Hanjun Liu (lhanjun@mail.sysu.edu.cn).

### Materials availability

This study did not generate new unique reagents.

### Data and code availability

• Data reported in this study will be shared by the lead contact upon reasonable request, subject to institutional agreements and ethical approvals.
• The original code for the CIA framework and data analyses is publicly available at https://github.com/zhaoshuhzi/CIA-IFG.
• Any additional information required to reanalyze the data reported in this study is available from the lead contact upon reasonable request.

## Acknowledgments

This study was funded by grants from the 10.13039/501100012166National Key R&D Program of China (no. 2025YFF0518000), the 10.13039/501100001809National Natural Science Foundation of China (nos. 82472600, 62271477, 82372558, 82372556, 82302848, 82302859, 82402971, 82503054), Shenzhen Science and Technology Program (nos. JCYJ20220818101217037, JCYJ20220818102800001), Shenzhen Medical Research Fund (C2401001), and The Science and Technology Planning Project of Guangdong Province (no. 2023B1212060018). Dr. Tingni Li is supported by InnoHK initiative and the Hong Kong Special Administrative Region Government.

## Author contributions

Y.C.: writing – original draft, investigation, and formal analysis; Q.G.: writing – original draft, investigation, and formal analysis; S.Z.: writing – original draft and formal analysis; T.L.: investigation and formal analysis; X.W.: formal analysis and data curation; D.L.: formal analysis and data curation; G.D.: formal analysis and data curation; X.Z.: formal analysis and data curation; X.C.: project administration and supervision; L.W.: project administration and supervision; P.L.: writing – review and editing, supervision, and project administration; N.Y.: writing – review and editing, conceptualization, supervision, and project administration; H.L.: writing – review and editing, conceptualization, supervision, and project administration.

## Declaration of interests

The authors declare no competing interests.

## STAR★Methods

### Key resources table

**Table:** 

REAGENT or RESOURCE	SOURCE	IDENTIFIER
**Deposited data**		

CIA analysis code	This paper	https://github.com/zhaoshuhzi/CIA-IFG

**Software and algorithms**		

G∗Power 3.1	Heinrich Heine University Düsseldorf	https://www.psychologie.hhu.de/arbeitsgruppen/allgemeine-psychologie-und-arbeitspsychologie/gpower
Visor 2.0	ANT Neuro	https://www.ant-neuro.com/products/visor2
Max/MSP 6.1	Cycling ’74	https://cycling74.com/downloads/older
LabChart 7.0	ADInstruments	https://www.adinstruments.com/labchart
NetStation 4.5	Electrical Geodesics Inc.	https://www.egi.com/
IGOR Pro 6.0	WaveMetrics Inc.	https://www.wavemetrics.com/downloads
Praat	Boersma	https://www.fon.hum.uva.nl/praat/
SPSS 20.0	IBM	https://www.ibm.com/products/spss-statistics
Brainstorm 3.2	University of Southern California and McGill University	https://neuroimage.usc.edu/brainstorm/
OpenMEEG	INRIA Sophia-Antipolis	https://openmeeg.github.io/

**Other**		

MRI scanner (3 T)	Siemens	Verio
TMS stimulator	YIRUIDE Co.	CCY-I
Optical motion tracking system	Northern Digital Inc.	Polaris Spectra
Dynamic microphone	Takstar Inc.	DM2200
Pitch-shifting processor	Eventide	Eclipse Harmonizer
Audio interface	MOTU	UltraLite-mk3 FireWire
Headphone amplifier	ICON	NeoAmp
Insert earphones	Etymotic Research Inc.	ER-1
A/D converter	ADInstruments	PowerLab ML880
Geodesic Sensor Net	Electrical Geodesics Inc.	N/A
EEG amplifier	Electrical Geodesics Inc.	Net Amps 300

### Experimental model and study participant details

#### Participants

An *a priori* power analysis using G∗Power 3.1 indicated that 20 participants were required for a repeated-measures analysis of variance (RM-ANOVA) across the three stimulation conditions, assuming an effect size of f = 0.30 based on previous studies,^30^^,^^31^^,^^35^ with α = 0.05, and power = 0.80. Twenty-four participants (12 females and 12 males; mean age: 22.13 ± 1.96 years) were recruited for the present study. All participants were right-handed, native-Mandarin speakers who were college students from Sun Yat-sen University. They were included in the experiment with the following criteria: no pregnancy; no intake of neuropsychiatric drugs; no implanted medical device; no experience of musical training; no history of speech, language, hearing, and neurological disorders. Three participants were excluded because of their failure to complete all three sessions (*n* = 1) or poor quality of EEG data (*n* = 2), leading to a final sample of 21 participants (10 females and 11 males; mean age: 22.19 ± 2.06 years) for statistical analyses. Written informed consent was obtained from all participants, and the research protocol was approved by the Institutional Review Board of The First Affiliated Hospital of Sun Yat-sen University (Approval No. 2021-885) in accordance with the Code of Ethics of the World Medical Association (Declaration of Helsinki).

### Method details

#### Transcranial magnetic stimulation

As a prerequisite for neuronavigated TMS, all participants underwent structural MRI scanning (Siemens Verio 3-T scanner, Germany). A high-resolution T1-weighted magnetization-prepared rapid gradient-echo (MPRAGE) sequence was used during the scanning with the following parameters: repetition time (TR) = 2300 ms, echo time (TE) = 2.19 ms, slice thickness = 1 mm, flip angle = 9°, field of view (FOV) = 256 × 256 mm^2^.

Magnetic stimulation was administered using a CCY-I TMS instrument (YIRUIDE Co., Wuhan, China) equipped with a 7-cm outer-diameter figure-of-eight coil. At the beginning of each experimental session, the motor hotspot corresponding to the right first dorsal interosseous (FDI) muscle was identified over the left primary motor cortex as the scalp location eliciting the largest and most consistent motor-evoked potentials (MEPs). Active motor threshold (AMT) was then determined as the minimum stimulation intensity that evoked MEPs ≥50 μV in at least 5 of 10 trials during mild voluntary contraction of the FDI (20% of maximal force).^66^ Stimulation intensity for cTBS was set to 80% of the session-specific AMT. Then, each participant underwent active cTBS over the left aIFG, active cTBS over the left pIFG, or sham cTBS over either left aIFG or left pIFG in a counterbalanced order on separate days at least 7 days apart. A standard cTBS protocol of 600 pulses was applied, with three pulses at 50 Hz every 200 ms for 40 s.^29^

Neuronavigation software (Visor 2.0, ANT Neuro, Netherlands) and a Polaris Spectra motion tracking system (NDI, Canada) were used to identify the target site and monitor coil position throughout the experiment. Each participant’s T1-weighted structural MRI was co-registered with the participant’s head in the neuronavigation system. The stimulation sites were defined using previously established mean MNI coordinates for the left anterior IFG (aIFG; x, y, z = −45, 27, 12 mm) and posterior IFG (pIFG; x, y, z = −47, 6, 21 mm).^67^^,^^68^ The group-level MNI coordinates were transformed into each participant’s native anatomical space using the inverse normalization transformation derived from the individual structural MRI. The aIFG target was located in the anterior IFG/pars triangularis region, whereas the pIFG target was located in the posterior IFG/pars opercularis region. The center-to-center Euclidean distance between the two group-level target coordinates was approximately 22.9 mm, reflecting an anterior-posterior separation within the left IFG. The resulting native-space target locations were inspected with reference to individual gyral and sulcal anatomy and used to guide subregional stimulation of the left IFG. For active stimulation, the center of the coil was aligned with the target site and the coil plane was placed tangent to the scalp. For sham stimulation, the coil plane was positioned perpendicular to the target site with the coil edge touching the scalp. Sham stimulation was applied over the left aIFG for half of the participants or over left pIFG for the other half.

#### Vocal production task

All participants performed a vocal production task based on the frequency-altered feedback (FAF) paradigm^4^ following active or sham cTBS over the target site. To minimize the interval between stimulation and task recording, cTBS and EEG recording were performed in the same experimental room and the EEG recording system was prepared before cTBS. Immediately after stimulation, the EEG cap was fitted, electrode impedances and signal quality were checked, and the FAF task was initiated within 10 minutes and completed within 25 minutes. The participants were instructed to vocalize the vowel sound /u/ at their habitual pitch and loudness levels when a blue light appeared on the computer monitor. Each vocalization lasted 6 s, during which the voice was unexpectedly pitch-shifted upward or downward by 200 cents (100 cents = 1 semitone) for 200 ms. Five pitch perturbations were presented in a pseudo-randomized manner during each vocalization, with the timing of the first perturbation randomly drawn from a rectangular distribution between 500 and 1000 ms after vocal onset and the inter-stimulus intervals for subsequent perturbations randomly drawn from a rectangular distribution between 700 and 900 ms. Prior to initiating the next vocalization, the participants were required to take a 5-s break to avoid vocal fatigue. Each participant produced 40 consecutive vocalizations for each stimulation session, leading to a total of 200 trials that included 100 trials with +200 cents perturbations and 100 trials with −200 cents perturbations. Across the three stimulation sessions, the vocal production experiment was conducted with the same parameters.

#### Data recording

The FAF-based vocal production experiment was conducted in a sound-attenuated booth. Voice signals were picked up by a dynamic microphone (model DM2200, Takstar Inc.) and sent to an Eventide Eclipse Harmonizer through a MOTU Ultralite Mk3 Firewire audio interface. A custom-developed Max/MSP software program (v.6.1 by Cycling ’74, CA) was used to control the Harmonizer for pitch-shifting the voice signals and to generate TTL pulses marking the onset of each perturbation. The pitch-shifted voice signals were amplified by an ICON NeoAmp headphone amplifier (Middleton, WI) and played back to participants through insert earphones (ER-1; Etymotic Research Inc.). The original voice signals, pitch-shifted feedback, and TTL pulses were digitized at 10 kHz by a PowerLab A/D converter (model ML880, ADInstruments) and recorded using LabChart software (v.7.0 by ADInstruments).

While participants performed the FAF-based vocal production task, EEG signals were recorded using a 64-electrode Geodesic Sensor Net connected to a Net Amps 300 amplifier (Electrical Geodesics Inc., Eugene, OR). The EEG signals across all channels referenced to the vertex (Cz) were recorded at 1 kHz using NetStation software (v.4.5, Electrical Geodesics Inc.). The impedance levels of individual electrodes were maintained below 50 kΩ throughout the recording in accordance with high-input impedance amplifier standards.^69^ The TTL pulses were sent to the EEG recording system via a Deutsches Institut für Normung (DIN) sync cable to ensure temporal alignment between voice and EEG signals.

#### Data analyses

A custom-developed software program (IGOR PRO, v.6.0 by WaveMetrics Inc.) was used to measure the vocal responses to pitch perturbations. First, the voice fundamental frequency (*f*_o_) contour in Hertz was extracted from the voice signals using Praat software^70^ and converted to the cents scale with the following formula: cents = 100 × (12 × log_2_[*f*_o_/reference]) (reference = 195.997 Hz [G3 note]). Then, the *f*_o_ contour in cents was segmented into epochs of −100 to +700 ms relative to the perturbation onset. All segmented trials were visually inspected on a trial-by-trial level to exclude those trials that contained artifacts such as unexpected vocal interruptions or signal processing errors. Finally, the artifact-free trials were averaged and baseline-corrected (−100 ms to 0) to generate an overall vocal response for each condition. The magnitude and latency of a vocal response were defined as the peak value in cents of the voice *f*_o_ contour and the corresponding time in milliseconds, respectively.

The EEG data were analyzed offline using NetStation software to extract the ERPs to pitch perturbations. First, the EEG signals were band-pass filtered between 1 and 20 Hz and segmented into epochs of −200 to +500 ms relative to the perturbation onset. An artifact rejection procedure was then applied, which rejected any trials whose voltage values exceeded ±55 μV of the moving average over an 80-ms window. Individual electrodes containing artifacts in more than 20% of the epochs would be marked bad in data-quality screening, and files containing more than 10 bad channels would be discarded from further analysis. The two participants excluded for poor EEG data quality failed to meet this criterion, and all datasets retained for ERP analysis and source localization satisfied it. Additional trial-by-trial visual inspection was performed to ensure that bad trials were appropriately rejected. For ERP averaging, the mean proportions of artifact-free trials retained across all perturbation trials were 82%, 81%, and 84% for the aIFG, pIFG, and sham conditions, respectively. Finally, artifact-free trials were re-referenced to the average of the electrodes on each mastoid^8^^,^^71^^,^^72^ and averaged with baseline correction (−200 to 0 ms) to generate an overall ERP response. Three regions of interest (ROIs) comprising 24 electrodes were chosen for statistical analysis: frontal area (AF3, AFz, AF4, F5, F3, F1, Fz, F2, F4, F6), fronto-central area (FC5, FC3, FC1, FCz, FC2, FC4, FC6), and central area (C5, C3, C1, Cz, C2, C4, C6). These ROIs were defined *a priori* based on previous studies showing that N1 and P2 responses to pitch perturbations are prominent over frontal, fronto-central, and central regions.^30^^,^^32^^,^^73^ The amplitudes and latencies of the N1 and P2 components were extracted from the averaged ERP response for each ROI. N1 amplitude was defined as the most negative peak within 80–180 ms after perturbation onset, and P2 amplitude was defined as the most positive peak within 160–280 ms after perturbation onset. The corresponding latencies were defined as the time points of these peaks.

In addition, a Global Dissimilarity (DISS) analysis^58^ was performed to further examine whether ERP effects were accompanied by changes in scalp-field configuration. DISS quantifies the topographic difference between two scalp electric fields after normalization by global field power, providing a strength-independent measure of response topography. DISS values were computed from 64-channel ERP maps for three pairwise stimulation contrasts (aIFG-sham, pIFG-sham, and aIFG-pIFG) within the N1 (80–180 ms) and P2 (160–280 ms) time windows for upward and downward pitch perturbations.

#### Causal inference analysis

To characterize stimulation-related patterns of source-space network organization supporting auditory-vocal integration, we proposed a CIA framework that integrated source-localized EEG data with individualized anatomical information to estimate directed dependencies among reconstructed cortical regions following cTBS over the left aIFG and pIFG (see Figure 1). Network analyses were performed separately for three pairwise stimulation contrasts: aIFG versus sham, pIFG versus sham, and aIFG versus pIFG. This framework consisted of three stages: (1) network construction, (2) model assessment, and (3) counterfactual estimation and robustness evaluation (code available at https://github.com/zhaoshuhzi/CIA-IFG). A detailed implementation of the CIA framework and robustness analyses is provided in Methods S1.

1) To provide source-level inputs for Bayesian network construction,^74^ ERP components (N1 and P2) were source-localized using Brainstorm software (v3.2), with forward models constructed via the boundary element method (BEM).^75^ Individual structural MRI data were co-registered with electrode positions using a mutual information algorithm.^76^ Source estimation employed minimum-norm estimation (MNE),^77^ with a regularization parameter corresponding to a signal-to-noise ratio (SNR) of 3. A distributed cortical source model was used, in which approximately 15,000 dipolar sources were distributed over the reconstructed cortical surface, with one source placed at each cortical vertex. The orientation of each source was fixed perpendicular to the local cortical surface. Thus, the estimated source activity reflected cortical current amplitudes along the surface-normal direction. Source-level activity was estimated within the same time windows used for ERP quantification, namely 80–180 ms for N1 and 160–280 ms for P2 after perturbation onset. Further details on source reconstruction and regional node definition are provided in Methods S1.

To assess the robustness of the CIA findings to inverse-solution choice, we repeated source reconstruction using weighted minimum-norm estimation (wMNE), standardized low-resolution brain electromagnetic tomography (sLORETA), and local autoregressive average (LAURA). The complete source-reconstruction and CIA pipeline was repeated for the aIFG-pIFG, aIFG-sham, and pIFG-sham contrasts. Cross-inverse robustness was assessed using source-map spatial correlations, node- and edge-level Jaccard overlap, and consensus-network recurrence. Details of the inverse-solution parameters and cross-inverse robustness analyses are provided in Methods S1.

Source-level cortical activity was parcellated using the Destrieux atlas, and a Bayesian network was constructed using a directed acyclic graph and a conditional probability distribution^74^ for each stimulation condition. For each participant and stimulation condition, vertex-level source amplitudes were averaged within each atlas-defined cortical region to obtain regional source-space time series. Each node in the network represented an atlas-defined cortical region, while directed edges captured model-estimated probabilistic dependencies. Directed transfer function (DTF) analysis^78^ was first used to estimate an initial weighted directed connectivity structure from regional source-space signals. This initial structure was subsequently optimized using Bayesian network structure learning, the Bayesian Dirichlet equivalent uniform (BDeu) scoring function,^79^ hierarchical clustering,^80^ and the Non-combinatorial Optimization via Trace Exponential and Augmented lagRangian for Structure learning (NOTEARS) algorithm.^81^ NOTEARS was used to reduce redundant edges, reciprocal connections, and feedback loops during graph-structure learning. Detailed descriptions of DTF-based initial connectivity estimation and Bayesian network/NOTEARS-based graph optimization are provided in Methods S1.

2) The optimized Bayesian network was assessed using structural equation modeling (SEM).^82^ SEM was used to evaluate whether the optimized network structure was consistent with the observed covariance structure and to compare path coefficients across stimulation conditions. The fit of each model was quantified using conventional SEM fit indices, and stimulation-induced differences were evaluated by comparing path coefficients across conditions. A Naive Bayes classifier^83^ was then applied to model-derived features to assess whether the optimized network contained condition-discriminative information (e.g., aIFG vs. sham; pIFG vs. sham) and to generate a discriminant matrix for comparing stimulation-specific network patterns. These procedures were used to assess model consistency and condition discrimination within the present dataset. Classifier comparison and NOTEARS ablation analyses were further performed to evaluate the discriminative performance of CIA-derived network features (see Tables S1 and S2). Detailed implementation of Bayesian-network-based model assessment, classifier comparison, and NOTEARS ablation is provided in Methods S1.
3) To test network stability, we employed a counterfactual stability testing procedure. This involved contrasting observed network patterns under active stimulation with estimated network patterns under sham stimulation. The Time Series Models with Independent Noise (TiMINo) algorithm^84^ was used to compare individual- and group-level networks across stimulation conditions. Robustness was evaluated by calculating Euclidean distances between discriminant matrices and by comparing the covariance structures of individual networks. In addition, Granger causality analysis^85^ was used as a complementary temporal-dependency analysis to assess directed temporal dependencies between cortical regions, with refinement using the partial coherence technique to estimate direct connections. Before Granger modeling, regional source-space time series were baseline-corrected, detrended, and z-scored. Stationarity was assessed using the augmented Dickey–Fuller test, model order was selected using the Akaike information criterion, and model adequacy was evaluated using residual whiteness. The autoregressive model was applied to regional source-space time series extracted from predefined ERP component windows (N1 and P2). Finally, counterfactual stability estimation was performed by comparing source-space signals from active stimulation with those from sham stimulation to characterize stimulation-related changes in model-inferred directed functional networks that support vocal pitch regulation. Directed-edge stability was additionally assessed using participant-level recurrence and 2,000 participant-resampled bootstrap iterations, with detailed procedures for counterfactual stability testing, temporal-dependency analyses, and edge-stability assessment provided in Methods S1.

### Quantification and statistical analysis

The normality of the vocal and ERP responses was verified using Kolmogorov-Smirnov tests. All data were analyzed using RM-ANOVA in SPSS (v.20.0). The peak magnitudes and times of vocal responses were subjected to two-way RM-ANOVAs with within-subject factors of stimulation session (left aIFG, left pIFG, and sham) and perturbation direction (+200 vs. −200 cents). The amplitudes and latencies of the ERP responses (N1 and P2) were subjected to three-way RM-ANOVAs with within-subject factors of stimulation session, perturbation direction, and electrode site (frontal, fronto-central, and central). Subsidiary RM-ANOVAs were performed for any significant higher-order interactions, and Bonferroni correction for multiple comparisons was applied for post-hoc analyses. DISS values for the N1 and P2 components were analyzed using separate one-way RM-ANOVAs for each perturbation direction, with contrast type (aIFG-sham, pIFG-sham, and aIFG-pIFG) as the within-subject factor. To control for multiple DISS comparisons, significant main effects were followed by Bonferroni-adjusted pairwise comparisons among contrasts. The Greenhouse-Geisser correction for multiple degrees of freedom was used to correct probability values when the assumption of Mauchly’s test of sphericity for homogeneity of variance was violated. Effect sizes indexed by partial η^2^ ($\eta_{p}^{2}$) were calculated to quantify the proportion of variance. All inferential analyses included *n* = 21 participants. Statistical details, including test statistics, degrees of freedom, *p* values, and effect sizes, are reported in the Results and figure legends. Measures of central tendency and dispersion are defined in the corresponding figure legends. The study was not designed or powered to test sex-related differences in cTBS effects; sex was therefore not included as a factor in the statistical analyses.
