# Supplementary material for: Probing the role of left inferior frontal gyrus in vocal motor control: Evidence from TMS and causal inference analysis
Source: iScience. 2026 Oct 8;29(11):117790. doi: 10.1016/j.isci.2026.117790 (PMC13677422; doi:10.1016/j.isci.2026.117790)
Supplement: Supplementary file 1 [file mmc1.pdf]

iScience, Volume *29*

Supplemental information

Probing the role of left inferior frontal gyrus

in vocal motor control: Evidence from TMS

and causal inference analysis

Yichen Chang, Qifan Guo, Shuzhi Zhao, Tingni Li, Xiuqin Wu, Dongxu Liu, Guangyan Dai, Xiaoxia Zhu, Xi Chen, Lan Wang, Peng Liu, Nan Yan, and Hanjun Liu

Supplementary information

Figure S1. Cross-method robustness of source-level patterns across stimulation contrasts. Source-level statistical maps were reconstructed using minimum-norm estimation (MNE), weighted minimum-norm estimation (wMNE), standardized low-resolution brain electromagnetic tomography (sLORETA), and local autoregressive average (LAURA). The upper row shows the direct aIFG-pIFG contrast, the middle row shows the aIFG-sham contrast, and the lower row shows the pIFG-sham contrast.

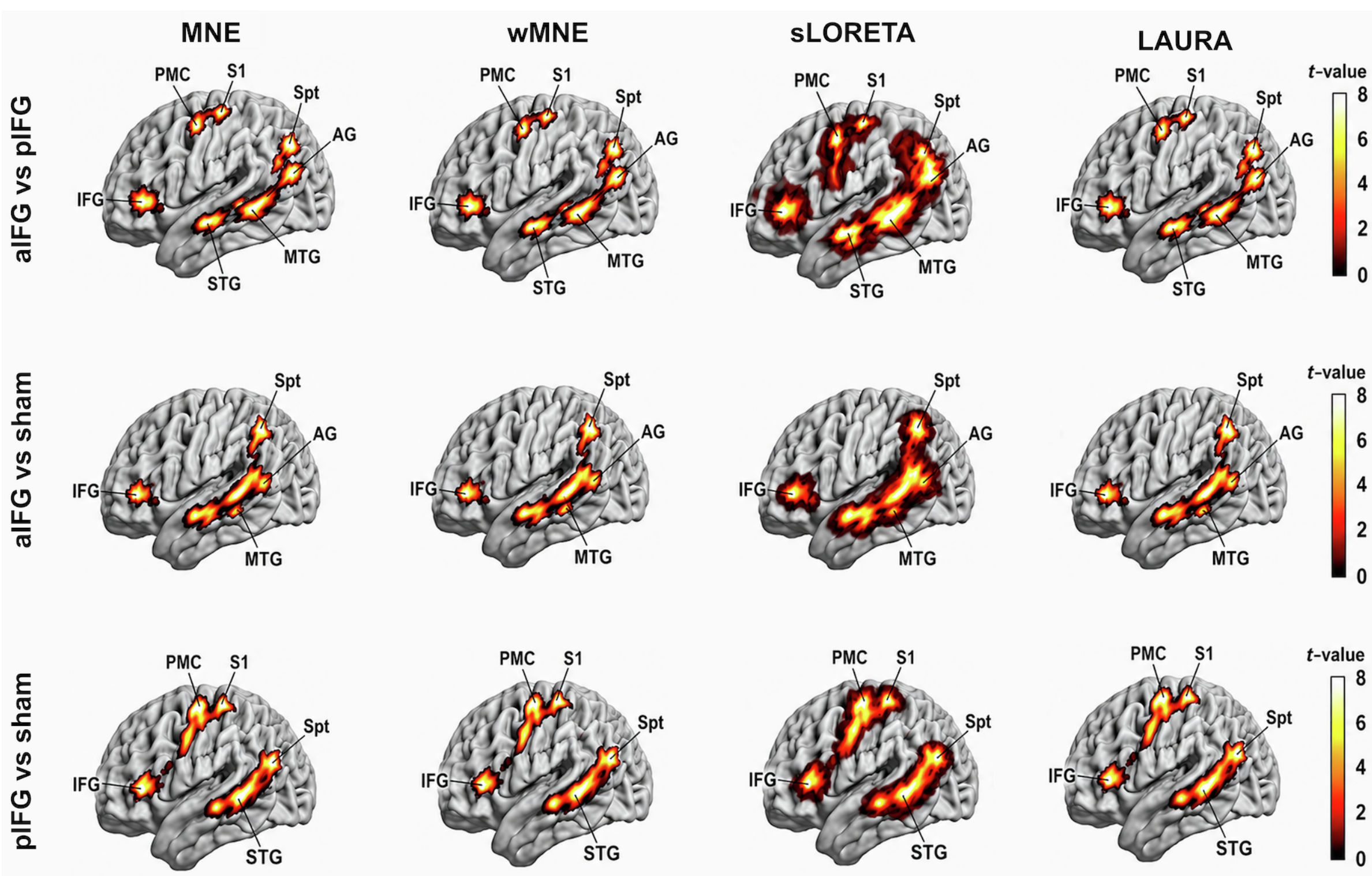

Figure S2. Cross-method consensus networks for the three stimulation contrasts. Consensus CIA-derived directed networks were constructed across MNE, wMNE, sLORETA, and LAURA for (A) aIFG-pIFG, (B) aIFG-sham, and (C) pIFG-sham. Red arrows indicate directed edges recovered with the same direction in all four inverse solutions (4/4), whereas orange arrows indicate directed edges recovered in three of four inverse solutions (3/4). L and R indicate the left and right hemispheres, respectively.

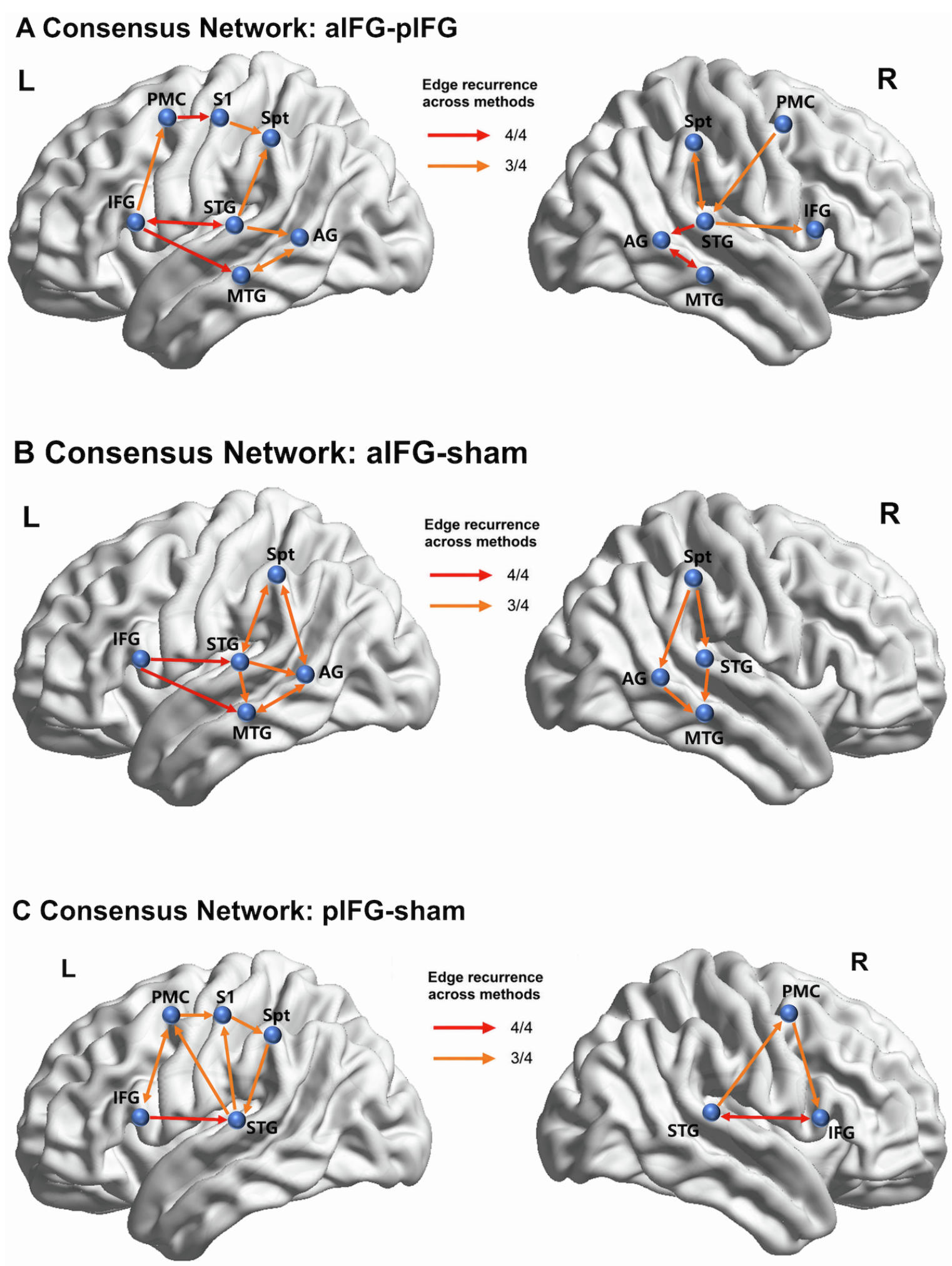

Figure S3. Quantitative convergence of source maps and network topology across inverse solutions. Left: Pairwise spatial correlations between source-level statistical maps reconstructed using MNE, wMNE, sLORETA, and LAURA for the aIFG-pIFG, aIFG-sham, and pIFG-sham contrasts. Correlation coefficients ranged from 0.69 to 0.82 for aIFG-pIFG, 0.72 to 0.86 for aIFG-sham, and 0.70 to 0.84 for pIFG-sham. Right: Jaccard indices quantifying cross-inverse overlap of network nodes and directed edges. Node-overlap indices were 0.74, 0.78, and 0.81, and directed-edge overlap indices were 0.61, 0.65, and 0.69 for the aIFG-pIFG, aIFG-sham, and pIFG-sham contrasts, respectively. Bars represent the mean pairwise Jaccard index across the six inverse-solution pairs; error bars represent the SEM across these pairwise comparisons.

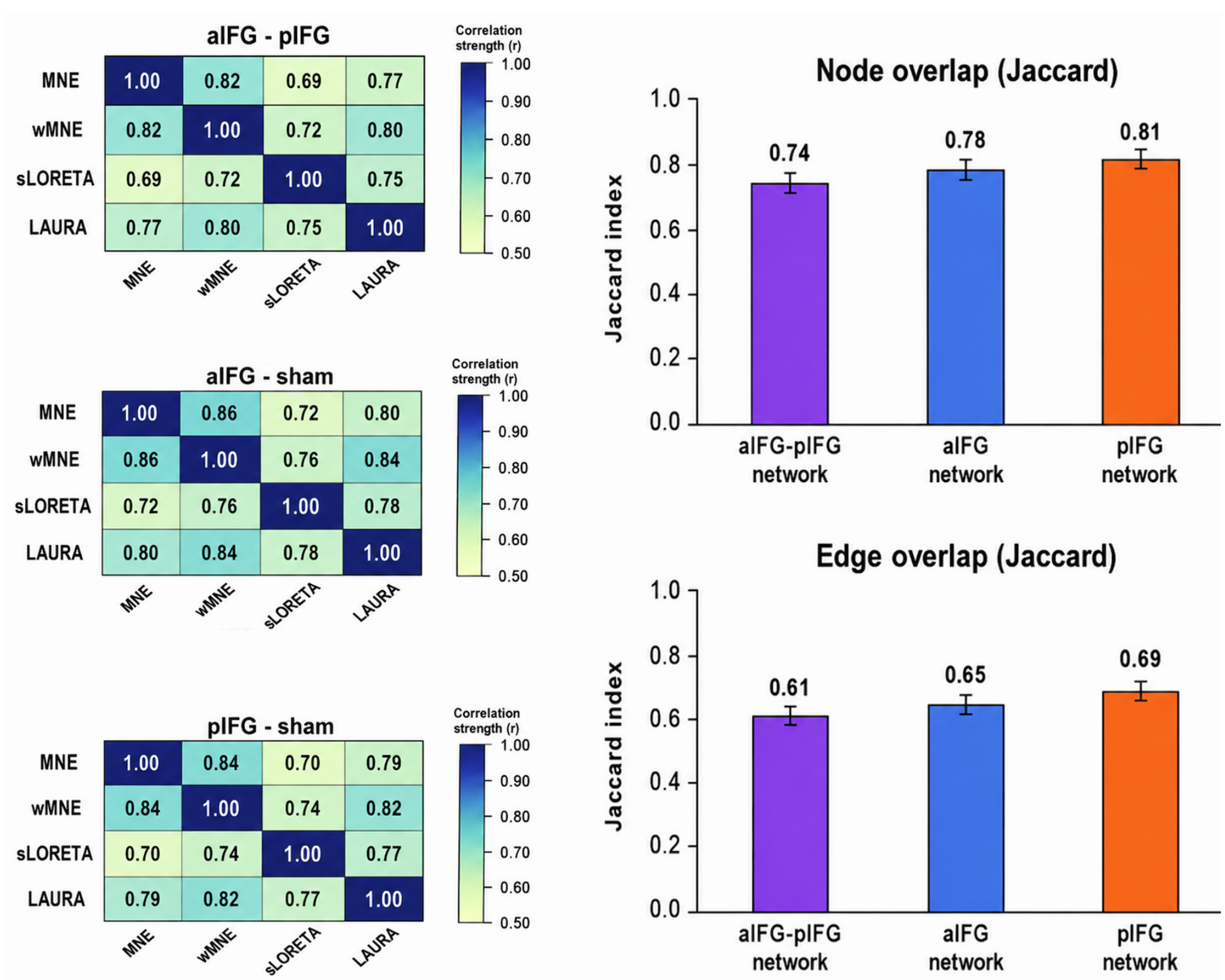

Table S1. Ablation results of CIA model across different algorithms to determine the effects of cTBS over the left aIFG on the N1 and P2 responses relative to sham stimulation.

Left aIFG-sham Classifier	Accuracy				Precision				Recall				F1 score			
	NOTEARS		No-NOTEARS		NOTEARS		No-NOTEARS		NOTEARS		No-NOTEARS		NOTEARS		No-NOTEARS	
	N1	P2	N1	P2	N1	P2	N1	P2	N1	P2	N1	P2	N1	P2	N1	P2
Nearest Neighbors	0.515837	0.515837	0.561086	**0.606335**	0.506088	0.566088	0.600213	**0.675991**	0.515837	0.515837	0.561086	**0.606335**	0.511077	0.511077	0.527206	**0.571355**
Linear SVM	**0.875928**	0.819005	0.647059	0.642534	**0.88434**	0.868255	0.646846	0.650021	**0.875928**	0.819005	0.647059	0.642534	**0.875139**	0.814001	0.64674	0.640373
RBF SVM	0.80543	**0.832579**	0.514163	0.514163	0.861207	**0.875597**	0.504414	0.504414	0.80543	**0.832579**	0.514163	0.524163	0.799095	**0.828703**	0.505887	0.515887
Gaussian Process	**0.898552**	0.841629	0.561086	0.58371	**0.898736**	0.87883	0.572549	0.6094	**0.898552**	0.841629	0.561086	0.58371	**0.898569**	0.836684	0.552135	0.566339
Decision Tree	**0.885928**	0.800905	0.837104	0.755656	**0.891839**	0.833843	0.857841	0.75846	**0.885928**	0.800905	0.837104	0.755656	**0.885761**	0.797106	0.835444	0.755506
Random Forest	0.773756	0.764706	0.723982	**0.78733**	0.800573	**0.806669**	0.737954	0.788224	0.773756	0.764706	0.723982	**0.78733**	0.770002	0.758202	0.721422	**0.787374**
Neural Net	**0.898552**	0.877828	0.683258	0.624434	**0.898552**	0.881223	0.684948	0.633852	**0.898552**	0.877828	0.683258	0.624434	**0.898552**	0.875476	0.683206	0.620951
AdaBoost	**0.903801**	0.884027	0.804027	0.810452	**0.90437**	0.881496	0.827003	0.810527	**0.903801**	0.884027	0.814027	0.820452	**0.903768**	0.89344	0.823995	0.810416
Naive Bayes	0.896425	**0.90095**	0.828054	0.832579	0.896796	**0.901116**	0.82826	0.832968	0.896425	**0.90095**	0.828054	0.832579	0.896429	**0.900952**	0.82809	0.83262
QDA	0.864253	**0.886878**	0.588235	0.59276	0.893979	**0.898302**	0.605624	0.608138	0.864253	**0.886878**	0.588235	0.59276	0.862315	**0.885829**	0.577488	0.583903

NOTEARS indicates models with NOTEARS-based graph refinement; No-NOTEARS indicates models without NOTEARS-based graph refinement. Bold values indicate the best performance within each metric comparison. SVM, support vector machine; RBF, radial basis function; QDA, quadratic discriminant analysis.

Table S2. Ablation results of CIA model across different algorithms to determine the effects of cTBS over the left pIFG on the N1 and P2 responses relative to sham stimulation.

Left pIFG-sham Classifier	Accuracy				Precision				Recall				F1 score			
	NOTEARS		No-NOTEARS		NOTEARS		No-NOTEARS		NOTEARS		No-NOTEARS		NOTEARS		No-NOTEARS	
	N1	P2	N1	P2	N1	P2	N1	P2	N1	P2	N1	P2	N1	P2	N1	P2
Nearest Neighbors	0.515837	0.515837	0.561086	**0.579186**	0.506088	0.506088	0.561217	**0.602848**	0.515837	0.515837	0.561086	**0.579186**	0.511077	0.511077	0.56114	**0.562439**
Linear SVM	**0.656109**	0.624434	0.556561	0.624434	**0.798927**	0.788497	0.560416	0.624337	**0.656109**	0.624434	0.556561	0.624434	0.615197	0.569423	0.554759	**0.624373**
RBF SVM	0.850679	**0.873303**	0.524163	0.534163	0.885876	**0.899581**	0.504414	0.504414	0.850679	**0.873303**	0.514163	0.524163	0.848019	**0.871764**	0.515887	0.525887
Gaussian Process	0.515837	0.515837	0.524887	**0.579186**	0.506088	0.506088	0.525314	**0.584717**	0.515837	0.515837	0.524887	**0.579186**	0.511077	0.511077	0.525004	**0.576642**
Decision Tree	0.638009	0.60181	0.746606	**0.755656**	0.755342	**0.775279**	0.758307	0.759535	0.638009	0.60181	0.746606	**0.755656**	0.597543	0.518204	0.744886	**0.755356**
Random Forest	0.638009	**0.81448**	0.764706	0.742081	0.745306	**0.863553**	0.779314	0.756881	0.638009	**0.81448**	0.764706	0.742081	0.600038	**0.806634**	0.762729	0.739689
Neural Net	0.751131	**0.764706**	0.615385	0.61991	0.832124	**0.838412**	0.616621	0.620167	0.751131	**0.764706**	0.615385	0.61991	0.732271	**0.748791**	0.615385	0.619987
AdaBoost	0.877828	**0.907602**	0.882353	0.873303	0.891406	**0.916512**	0.882411	0.873498	0.877828	**0.907602**	0.882353	0.873303	0.876333	**0.90705**	0.88231	0.873329
Naive Bayes	0.9019	**0.92095**	0.78733	0.755656	0.902553	**0.921116**	0.787725	0.75588	0.9019	**0.92095**	0.78733	0.755656	0.901905	**0.920952**	0.787383	0.755706
QDA	0.882353	**0.918326**	0.570136	0.556561	0.905352	**0.910271**	0.570265	0.560416	0.882353	**0.918326**	0.570136	0.556561	0.881154	**0.918326**	0.570189	0.554759

NOTEARS indicates models with NOTEARS-based graph refinement; No-NOTEARS indicates models without NOTEARS-based graph refinement. Bold values indicate the best performance within each metric comparison. SVM, support vector machine; RBF, radial basis function; QDA, quadratic discriminant analysis.

Table S3. Participant-level recurrence and bootstrap occurrence of principal CIA-derived directed edges across stimulation contrasts.

Condition	Hemisphere	Directed edge	Participant recurrence	Recurrence (%)	Bootstrap occurrence (%)
**aIFG-sham**	Left	IFG → STG	18/21	85.7	87.4
	Left	IFG → MTG	17/21	81.0	83.1
	Left	STG → AG	16/21	76.2	79.6
	Right	Spt → STG	15/21	71.4	75.8
	Right	STG → MTG	15/21	71.4	72.9
**pIFG-sham**	Left	IFG → PMC	18/21	85.7	86.6
	Left	IFG → STG	17/21	81.0	84.2
	Left	STG → PMC	16/21	76.2	81.5
	Right	STG → PMC	15/21	71.4	76.4
	Right	IFG → STG	14/21	66.7	72.3
**aIFG-pIFG**	Left	IFG → MTG	16/21	76.2	79.6
	Left	STG → AG	15/21	71.4	75.8
	Right	Spt → STG	15/21	71.4	72.9
	Left	IFG → PMC	15/21	71.4	76.4
	Right	PMC → STG	14/21	66.7	72.3

Participant-level recurrence indicates the number and percentage of the 21 participants in whom each group-level directed edge was recovered with the same direction. Bootstrap occurrence indicates the percentage of 2,000 participant-resampled group networks in which the same directed edge was recovered. Results are reported separately for the aIFG-sham, pIFG-sham, and aIFG-pIFG networks.

Methods S1. Detailed implementation and robustness assessment of the causal inference analysis framework.

Overview of the CIA framework

This supplementary section provides a detailed description of the implementation of the causal inference analysis (CIA) framework used to characterize model-inferred directional network patterns during auditory-vocal integration. The CIA framework was applied to source-localized EEG activity and individualized anatomical information to construct, optimize, and assess source-space brain networks across the left aIFG, pIFG, and sham stimulations. The analysis included source reconstruction and regional node definition, DTF-based initial directed connectivity estimation, Bayesian network learning and NOTEARS-based graph optimization, within-dataset model assessment and NOTEARS ablation, model-based network stability and temporal-dependency analyses, cross-inverse robustness analyses, and participant-level and bootstrap assessment of directed-edge stability.

Source reconstruction and regional node definition

ERP responses were first reconstructed in cortical space to provide regional source inputs for network modeling. Source reconstruction was performed using Brainstorm software [1]. For each participant, an individualized head model was constructed from the T1-weighted structural MRI. EEG electrode positions were co-registered to the individual anatomy using a mutual information-based registration procedure [2]. The forward model was computed using a boundary element head

model [3], and source estimation was performed using distributed minimum-norm estimation (MNE), with a regularization parameter corresponding to a signal-to-noise ratio (SNR) of 3 [4]. The inverse solution was based on a distributed cortical source model rather than a small number of freely fitted equivalent current dipoles. Approximately 15,000 dipolar sources were defined on the reconstructed cortical surface, with one source at each cortical vertex. The orientation of each source was fixed perpendicular to the local cortical surface, yielding estimates of cortical current amplitudes along the surface-normal direction. Source reconstruction was performed on averaged ERP responses rather than on single-trial source estimates.

To evaluate the robustness of the source-space findings to the choice of inverse solution, source reconstruction was repeated using weighted minimum-norm estimation (wMNE), standardized low-resolution brain electromagnetic tomography (sLORETA), and local autoregressive average (LAURA). These methods were selected because they apply distinct constraints to the EEG inverse problem. wMNE applies depth weighting to reduce the superficial-source bias of conventional MNE; sLORETA standardizes current-density estimates to improve spatial comparability across cortical locations; and LAURA incorporates local spatial constraints among neighboring cortical sources.

Across the four inverse solutions, the same participant-specific MRI-derived BEM forward models, electrode co-registration, cortical source space (~15,000 vertices), source-orientation constraints, ERP time windows, and Destrieux parcellation were retained. Source reconstruction was

performed separately using each inverse solution, and the resulting regional source-space signals were separately entered into the same downstream CIA pipeline, including DTF-based connectivity estimation, Bayesian network learning, NOTEARS optimization, within-dataset SEM-based model assessment, and temporal-dependency analysis. The full analysis pipeline was repeated for the aIFG-pIFG, aIFG-sham, and pIFG-sham contrasts. Method-specific inverse settings were as follows: wMNE, with a depth-weighting exponent of 0.5 and a maximum depth-weighting factor of 10; sLORETA, with resolution-kernel standardization, an assumed SNR of 3, and noise-covariance regularization of 0.1; and LAURA, using local autoregressive spatial constraints with distance-dependent weighting and L-curve-based regularization.

The reconstructed cortical source activity was then parcellated according to the Destrieux atlas [5]. The network analysis included 14 regions: the bilateral IFG, STG, MTG, AG, PMC, S1, and Spt. Each node therefore represented an anatomically defined cortical region rather than a scalp electrode. For each participant and each stimulation condition, vertex-level source amplitudes were extracted within each region of interest and averaged across vertices to obtain regional source time series per node. These regional signals served as the regional source-activity variables entered into the subsequent directed network analysis. Source-space estimates were generated separately within the predefined N1 and P2 component windows used for scalp-level ERP quantification.

DTF-based initial directed connectivity estimation

For each stimulation condition, directed inter-regional connectivity was estimated in source space using the directed transfer function (DTF). DTF is a multivariate autoregressive-model-based frequency-domain method for estimating directional spectral dependencies among multiple electrophysiological signals [6, 7]. In the present analysis, DTF was applied to regional source-space signals rather than scalp electrode signals, generating an initial weighted directed adjacency matrix between atlas-defined cortical regions. The MVAR model order was selected using the Akaike information criterion (AIC) from candidate orders 1–20, yielding an order of 5. With 14 network nodes, 182 possible directed non-self edges were evaluated within each contrast. Edges were retained only when they exceeded a DTF threshold of 0.20 and survived Benjamini–Hochberg false discovery rate correction across the 182 candidate edges (FDR-adjusted $P < 0.05$). The resulting connectivity structure served as the basis for subsequent Bayesian network learning and NOTEARS-based graph optimization.

Bayesian network learning and NOTEARS-based graph optimization

The initial DTF-based connectivity structure was further refined using Bayesian network structure learning and NOTEARS-based graph optimization. Bayesian network modeling was used to characterize conditional dependencies among regional source-activity variables [8]. Within this framework, each node represented an atlas-defined cortical region, and each directed edge represented a conditional dependency between regional source signals. Network structure was evaluated using the Bayesian Dirichlet equivalent uniform (BDeu) scoring function [9]. To obtain a sparse directed acyclic graph (DAG) compatible with Bayesian network representation, we applied

NOTEARS (Non-combinatorial Optimization via Trace Exponential and Augmented Lagrangian for Structure learning) [10]. NOTEARS was used to reduce redundant edges, reciprocal connections, and cyclic dependencies during structure learning. After optimization, the directed graph was represented as a Bayesian network for each stimulation condition. Conditional probability distributions were used to characterize the dependency structure among regional source-activity variables under the aIFG, pIFG, and sham stimulation conditions.

Hierarchical clustering [11] was applied after source parcellation and network construction to group cortical regions according to similarity in regional source activity or conditional-dependency profiles related to N1/P2 responses. This step was used to facilitate interpretation of network organization and was not used to determine the number or location of source dipoles.

Within-dataset model assessment and NOTEARS ablation

The refined Bayesian network was assessed using structural equation modeling (SEM) [12]. SEM was used to examine whether the optimized network structure was consistent with the observed covariance structure among regional source-activity variables and to compare path coefficients across stimulation conditions. Model fit was quantified using conventional SEM fit indices. To evaluate whether the optimized network contained stimulation-discriminative information, a Naive Bayes classifier [13] was trained on network-derived features to distinguish active stimulation from sham stimulation, including aIFG versus sham and pIFG versus sham. The resulting discriminant matrices were used to characterize condition-related differences in network patterns.

To further characterize the within-dataset discriminative information contained in the CIA-derived network features, we conducted a classifier comparison and NOTEARS ablation analysis using features derived from the hierarchical clustering results. The analyses were performed separately for the aIFG-versus-sham and pIFG-versus-sham comparisons using N1- and P2-related source-space features. Ten classifiers were evaluated, including nearest neighbors, linear support vector machine (SVM), radial basis function SVM, Gaussian process, decision tree, random forest, neural network, AdaBoost, Naive Bayes, and quadratic discriminant analysis. To assess the contribution of NOTEARS-based graph refinement, classifier performance was compared between models with NOTEARS and models without NOTEARS.

Model performance was summarized using accuracy, precision, recall, and F1 score, with F1 score calculated as the harmonic mean of precision and recall. A hierarchical clustering parameter of 135 was used for the reported classifier comparison. Performance was compared between NOTEARS and No-NOTEARS models to characterize the contribution of NOTEARS-based graph refinement within the present dataset.

Counterfactual stability testing and temporal-dependency analyses

To assess stimulation-related network stability relative to the sham condition, we performed counterfactual stability testing. Specifically, network patterns observed under active stimulation were compared with model-estimated network patterns under the sham condition. The Time Series

Models with Independent Noise (TiMINo) algorithm was used to compare individual- and group-level networks across stimulation conditions [14]. Robustness was evaluated by calculating Euclidean distances between discriminant matrices and by comparing covariance structures of individual networks.

In addition, Granger causality analysis was used as a complementary temporal-dependency analysis to examine model-inferred directed temporal relationships between reconstructed cortical regions 15. Because source-localized ERP responses preserve time-resolved regional trajectories within the post-perturbation window, the autoregressive model tested whether activity in one reconstructed cortical region improved the temporal prediction of activity in another region. The analysis was performed on regional source-space time series extracted from Destrieux atlas-defined cortical regions within the N1 and P2 component windows. Before model fitting, regional source time series were baseline-corrected, detrended, and z-scored. Stationarity was assessed using the augmented Dickey–Fuller (ADF) test, model order was selected using the Akaike information criterion (AIC), and model adequacy was evaluated using residual whiteness. To reduce indirect connections, directed connectivity estimates were refined using partial coherence-related procedures. The downstream CIA procedures described above were applied identically to source-space signals reconstructed using each inverse solution. Cross-inverse comparisons of the resulting source maps and network structures are described in the Cross-inverse robustness analysis subsection.

Cross-inverse robustness analysis

Source-map similarity

To quantify the spatial consistency of source-level findings across inverse solutions, source-level statistical maps obtained using MNE, wMNE, sLORETA, and LAURA were compared separately for the aIFG-pIFG, aIFG-sham, and pIFG-sham contrasts. Pairwise Spearman spatial correlations were calculated across corresponding cortical vertices for each pair of inverse solutions. Higher correlation coefficients indicated greater spatial similarity between source patterns across different inverse methods.

Node- and edge-overlap analysis

Network-level convergence was quantified separately for network nodes and directed edges using the Jaccard index. For two sets A and B, the Jaccard index was defined as

$$J(A,B) = \frac{[A \cap B]}{[A \cup B]}$$

Node overlap was calculated from the sets of cortical regions identified in the CIA-derived networks, whereas edge overlap was calculated from the sets of directed connections between regions. Pairwise Jaccard indices were calculated across the four inverse solutions for each of the three contrasts.

Cross-method consensus networks

To identify network features that were consistently reproduced across inverse solutions, consensus networks were constructed separately for the aIFG-pIFG, aIFG-sham, and pIFG-sham contrasts.

Each directed edge was evaluated according to its recurrence across the four reconstructed CIA networks. Edges recovered with the same direction in all four inverse solutions were classified as 4/4 consensus edges, whereas edges recovered with the same direction in three of four solutions were classified as 3/4 consensus edges. Edges recovered in fewer than three inverse solutions were not included in the consensus-network visualization.

Participant-level and bootstrap directed-edge stability

To quantify the stability of CIA-derived directed edges across participants, the CIA network was estimated separately for each of the 21 participants using the same source reconstruction, regional parcellation, connectivity estimation, graph refinement, and edge-selection procedures as in the primary analysis. For each directed edge identified in the group-level aIFG-sham, pIFG-sham, or aIFG-pIFG network, participant-level recurrence was defined as the number of participants in whom the same directed edge was recovered with the same direction. Recurrence percentage was calculated as the recurrence count divided by 21 and multiplied by 100.

Bootstrap stability was further assessed using 2,000 participant-level resamples. For each bootstrap iteration, 21 participants were sampled with replacement from the original sample, and the group-level CIA network was reconstructed using the same analytical pipeline and edge-selection criteria. Bootstrap occurrence for each directed edge was defined as the percentage of bootstrap networks in which the same edge was recovered with the same direction. Participant-level recurrence and bootstrap occurrence for the principal directed edges are reported in Table S3.

Supplemental References

1. Tadel, F., Baillet, S., Mosher, J.C., Pantazis, D., and Leahy, R.M. (2011). Brainstorm: a user-friendly application for MEG/EEG analysis. Comput Intell Neurosci *2011*, 879716.
2. Maes, F., Collignon, A., Vandermeulen, D., Marchal, G., and Suetens, P. (1997). Multimodality image registration by maximization of mutual information. IEEE Trans Med Imaging *16*, 187-198.
3. Gramfort, A., Papadopoulo, T., Olivi, E., and Clerc, M. (2010). OpenMEEG: opensource software for quasistatic bioelectromagnetics. Biomed Eng Online *9*, 45.
4. Hamalainen, M.S., and Ilmoniemi, R.J. (1994). Interpreting magnetic fields of the brain: minimum norm estimates. Med Biol Eng Comput *32*, 35-42.
5. Destrieux, C., Fischl, B., Dale, A., and Halgren, E. (2010). Automatic parcellation of human cortical gyri and sulci using standard anatomical nomenclature. NeuroImage *53*, 1-15.
6. Kamiński, M.J., and Blinowska, K.J. (1991). A new method of the description of the information flow in the brain structures. Biol Cybern *65*, 203-210.
7. Kamiński, M., Ding, M., Truccolo, W.A., and Bressler, S.L. (2001). Evaluating causal relations in neural systems: granger causality, directed transfer function and statistical assessment of significance. Biol Cybern *85*, 145-157.
8. Suebnukarn, S., and Haddawy, P. (2006). A Bayesian approach to generating tutorial hints in a collaborative medical problem-based learning system. Artif Intell Med *38*, 5-24.
9. Suzuki, J. (2017). A theoretical analysis of the BDeu scores in Bayesian network structure

learning. Behaviormetrika *44*, 97-116.

10. Zheng, X., Aragam, B., Ravikumar, P.K., and Xing, E.P. (2018). Dags with no tears: Continuous optimization for structure learning. Adv Neural Inf Process Syst *31*.

11. Mammone, N., Ieracitano, C., Adeli, H., Bramanti, A., and Morabito, F.C. (2018). Permutation Jaccard Distance-Based Hierarchical Clustering to Estimate EEG Network Density Modifications in MCI Subjects. IEEE Trans Neural Netw Learn Syst *29*, 5122-5135.

12. Kline, R.B. (2023). Principles and practice of structural equation modeling, (Guilford publications).

13. Wolfson, J., Bandyopadhyay, S., Elidrisi, M., Vazquez-Benitez, G., Vock, D.M., Musgrove, D., Adomavicius, G., Johnson, P.E., and O'Connor, P.J. (2015). A Naive Bayes machine learning approach to risk prediction using censored, time-to-event data. Stat Med *34*, 2941-2957.

14. Peters, J., Janzing, D., and Schölkopf, B. (2013). Causal inference on time series using restricted structural equation models. Adv Neural Inf Process Syst *26*.

15. Seth, A.K., Barrett, A.B., and Barnett, L. (2015). Granger causality analysis in neuroscience and neuroimaging. J Neurosci *35*, 3293-3297.
